# Domain architecture of BAF250a reveals the ARID and ARM-repeat domains with implication in function and assembly of the BAF remodeling complex

**DOI:** 10.1371/journal.pone.0205267

**Published:** 2018-10-11

**Authors:** Sankaran Sandhya, Aditi Maulik, Malyasree Giri, Mahavir Singh

**Affiliations:** 1 Molecular Biophysics Unit, Indian Institute of Science, Bengaluru, India; 2 NMR Research Centre, Indian Institute of Science, Bengaluru, India; University of Washington, UNITED STATES

## Abstract

BAF250a and BAF250b are subunits of the SWI/SNF chromatin-remodeling complex that recruit the complex to chromatin allowing transcriptional activation of several genes. Despite being the central subunits of the SWI/SNF complex, the structural and functional annotation of BAF250a/b remains poorly understood. BAF250a (nearly 2200 residues protein) harbors an N-terminal DNA binding ARID (~110 residues) and a C-terminal folded region (~250 residues) of unknown structure and function, recently annotated as BAF250_C. Using hydrophobic core analysis, fold prediction and comparative modeling, here we have defined a domain boundary and associate a β-catenin like ARM-repeat fold to the C-terminus of BAF250a that encompass BAF250_C. The N-terminal DNA-binding ARID is found in diverse domain combinations in proteins imparting unique functions. We used a comparative sequence analysis based approach to study the ARIDs from diverse domain contexts and identified conserved residue positions that are important to preserve its core structure. Supporting this, mutation of one such conserved residue valine, at position 1067, to glycine, resulted in destabilization, loss of structural integrity and DNA binding affinity of ARID. Additionally, we identified a set of conserved and surface-exposed residues unique to the ARID when it co-occurs with the ARM repeat containing BAF250_C in BAF250a. Several of these residues are found mutated in somatic cancers. We predict that these residues in BAF250a may play important roles in mediating protein-DNA and protein-protein interactions in the BAF complex.

## Introduction

Chromatin remodeling complexes are specialized cellular machinery consisting of a number of proteins that work in concert, to remodel the nucleosome (histone-DNA complex) structure during various cellular processes such as transcription, replication, and repair leading to regulation of gene expression [1]. Both the histone modification enzymes and the ATP-dependent complexes play key roles in mediating this reorganization. While the former modify histones, the latter utilize ATP hydrolysis to drive the local disruption or alter association of histones with DNA. Such complexes, as observed in humans, yeast and drosophila typically involve as many as twelve to fifteen proteins [2,3]. Each protein is known to exist in more than one isoform, thus expanding the scope for the formation of a wide range of complexes that can function distinctly from each other. Indeed, it is suggested that the controlled expression of specific sub-units in various developmental stages is possibly a means to regulate the composition of individual complexes and their interactions with intended targets [4,5].

BAF250a (also known as ARID1A_HUMAN or P270) is a characteristic member of the ATP-dependent SWI/SNF chromatin remodeling complex in humans that is thought to recruit the complex to the chromatin through either protein-DNA or protein-protein interactions [6–8]. It is known to exist in the complexes in a mutually exclusive state with its paralogue BAF250b (or ARID1B_HUMAN) resulting in functionally distinct complexes: BAF-A (with BAF250a) or BAF-B (with BAF250b) SWI/SNF remodeling complexes [7,9–11]. Recently, several studies have revealed that BAF250a mutations are observed in a variety of cancers [8,12,13]. Depletion of BAF250b in BAF250a mutant cell lines resulted in the dissociation of the other subunits of the SWI/SNF complexes showing that BAF250a and BAF250b are a central component of these complexes [14]. Other protein subunits of the SWI/SNF complex include actin (ACTB), SMARCA2, SMARCA4/BRG1/BAF190A, ACTL6A/BAF53, ACTL6B/BAF53B, SMARCE1/BAF57, SMARCC1/BAF155, SMARCC2/BAF170, SMARCB1/SNF5/INI1, and one or more of SMARCD1/BAF60A, SMARCD2/BAF60B, or SMARCD3/BAF60C [2]. Although the major players of the complex are known, the nature of their interaction either with themselves or with their targets are not clearly established.

The structure and functional annotation of BAF250a/b (> 2200 residues long proteins) remains poorly understood. The folded regions and domain boundaries of these lengthy proteins have not been clearly defined. Likewise, their DNA-binding specificities and potential protein-protein interactions have not been studied systematically [15]. BAF250a/b contains two annotated folded domains: an N-terminal AT-rich interacting domain (ARID, ~110 residues) and a C-terminal domain of unknown function (DUF3518, ~ 256 residues) in PFAM, recently annotated as BAF250_C [15]. Presently, no other domain has been associated with the region interspersed between these two domains although functionally annotated sites such as GR-binding LXXL motifs, NES (nuclear export signal), NLS (nuclear localization signal), etc., have been identified in this regions.

As mentioned earlier, BAF250a is believed to recruit the SWI/SNF complexes to specific genes either through its interaction with DNA or binding to transcription factors, transcriptional co-activators and transcriptional co-repressors complexes [8,16–18]. The N-terminal ARID in BAF250a is primarily responsible for it’s interaction with DNA. The evidences so far suggest that BAF250a ARID interacts with DNA without any sequence preference [19–21], therefore questioning the recruitment of SWI/SNF complexes by BAF250a to the target genes *via* its interaction with specific DNA sequences. BAF250a has also been linked to nuclear hormone induced transcription and expression of cell-cycle regulators. Several nuclear hormone-binding LXXLL motifs and a GR (glucocorticoid receptor)-binding region have been mapped to the C-terminal region of the protein that contains the BAF250_C [8].

In this study, using sequence and hydrophobic core analysis, we predict the existence of folded regions along the sequence of BAF250a [22]. This includes the N-terminal ARID, the C-terminal region that encompasses the BAF250_C, and 3 other regions of at least 100 residues length. The occurrence of the ARID in multiple domain contexts, with a variety of partnering domains, motivated us to probe for sequence-specific signatures on the ARID that could drive or dictate its folding and functions, such as DNA and protein interactions in BAF250a/b. We performed a systematic study of the alignments of ARIDs in 11 major domain contexts to determine if such architecture-dependent sequence signatures exist. Significantly, in a domain architecture where ARID always co-occurs with BAF250_C (e.g. in BAF250a/b), we examined alignments of homologues of BAF250a/b that conserve the same domain organization and identified a set of conserved residues in ARID, which could be further classified into two distinct sets. One set constitutes conserved residues that are required for the maintenance of the ARID core, while the other set constitutes conserved but surface exposed residues that are unique to ARID when in association with BAF250_C. Likely, such residues that are uniformly conserved in all the homologues may have important functional roles in driving the interactions with other regions of BAF250a/b or other proteins in the SWI/SNF complex. Similarly, our analysis also reveals conserved and surface exposed residues in BAF250_C when it co-occurs with ARID (in BAF250a/b). Such selective conservation of residues unique to this combination of domains may have a bearing on the globularity of BAF250a and also mediate BAF250a association with other members of SWI/SNF complex *via* their interaction with the BAF250_C, which is predicted to adopt the ARM repeat fold typically involved in protein-protein interactions. Through motif searches, we also recognize potential interactions sites in BAF60B, BRG1, BAF53B implying that these may be the interacting partners of BAF250_C in the SWI/SNF complex. Further, we explored the distribution of somatic mutations reported in the BAF250a gene to predict the consequences of such mutations on the structures of the ARID and modelled structure of BAF250_C. Majority of these residues are surface exposed and therefore mutation of these residues can potentially lead to impairment of DNA and/or protein binding ability of BAF250a.

## Materials and methods

### Dataset

Homologues of the proteins containing the ARID were obtained by performing iterative jackhmmer searches [23] against the UniProt database (v.2017_03) at the EBI server [24]. Seed alignments of the ARID containing proteins from PFAM 31.0 were employed as an input to jumpstart the searches. Searches were performed for up to five iterations at E-value inclusion and reporting thresholds less than 0.0001 to identify 4414 proteins that contain the ARID domain. Domain assignments, using pfamscan [25], were performed for the identified hits to prune for false positives and to identity ARID containing proteins that co-occur with the C-terminal BAF250_C.

### Domain alignments of the ARID and BAF250_C

239 ARID sequences were found to co-occur with the BAF250_C in our dataset. To retain only full-length sequences, sequences that were truncated/fragments were excluded and the remaining sequences were aligned using command-line MUSCLE (v3.8.31) [26]. Care was taken to include homologues of known structure, where applicable, and a total of 210 sequences from diverse organisms were considered for the ARID dataset. Likewise, cognate sequences of 210 BAF250_C terminal domain were extracted and aligned using MUSCLE. Here again, fragments and truncated domains were excluded.

Once aligned, unrooted phylogenetic trees were derived for the two domains separately to compare the sequences, using the Neighbour-joining algorithm in Phylip with 1000 bootstraps [27].

### Domain contexts and derived architectures

The ARID family in PFAM is represented by 3169 sequences in the full alignment and 4414 proteins in the jackhmmer searches performed here. In all, based on domain assignments, these sequences are observed in 164 architectures or domain combinations, suggesting that the ARID occurs in diverse domain contexts. Careful examination of the various domain contexts showed that the same domains reappear in different orders or numbers in various domain contexts. For example, 13 sequences are observed to possess the JmjN, ARID, PHD, JmjC, zf-C5HC2, and three copies of PLU-1 (3X) domain combination while 17 sequences contain JmjN, ARID, PHD, JmjC, zf-C5HC2 and two copies of PLU-1 (2X) domains. Although the nature of the domains is identical in the two sets of sequences, the number of copies of the PLU-1 domains varies with the latter set containing two copies of the PLU-1 domains.

To evaluate if any sequence signals distinguish the various domain combinations, we extracted a subset of the 164 contexts of the ARID with a unique representation of domain combinations (S1 Table). In this way, we eliminated redundancies in the sequences representing the same domain combinations that differed in the number of copies of the individual domains in the dataset.

The eleven such unique domain contexts that were considered for further analysis are listed in S1 Table. As shown here, the number of sequences in each domain architecture varied. For instance there are nearly 1395 sequences in the domain context that contains only the ARID, while there are 239 sequences in the context that contain both the ARID and BAF250_C domains and only 6 sequences that contain the Myb_DNA-bind_2, ARID, Rap1_C domain combinations. To remove redundant sequences and restrict the analysis to a representative set of sequences in each architecture, we clustered the sequences within each architecture using blastclust from the NCBI–BLAST package [28], at 80% identity over 90% of the length of each sequence. Architectures with fewer sequences were not subjected to clustering to retain a minimal meaningful set for sequence comparisons. We also determined the average length of the ARID in each domain architecture and considered only those sequences that were +/-15 residues length-deviant from the average ARID length in each architecture. Although stringent, this excluded sequences that were fragmented or very long in each architecture.

### Generation of structure-guided multiple sequence alignments of ARIDs in each architecture

The regions corresponding to the ARID were extracted from each architecture and multiple sequence alignments were performed using the default options of command-line MUSCLE (v3.8.31) [26]. Conservation of residues in the alignment was derived based on identity, similarity and biochemical properties. The fingerprints derived for each architecture were compared using an alignment of the ARIDs across all architectures as a reference (data not shown, available on request). Since the structure of the ARID was available in some of the domain architectures, these were included in the cognate alignment, where applicable. Property and conservation-based fingerprints for each architecture were derived and visualized using Mview [29] and Espript [30].

### Preparation of ARID WT and V1067G mutant proteins and DNA for CD and NMR spectroscopy

ARID (residues 968–1143) of BAF250a was cloned in pET28a vector that imparts an N-terminal 6XHis-tag to protein. Site-directed mutagenesis was performed using Q5 mutagenesis kit to produce valine (V) to glycine (G) mutation at 1067 residue position in ARID. Wild type (WT) and mutant (V1067G) proteins were expressed in E. Coli BL21(DE3)-Rosetta cells using 1 mM IPTG for induction at 20°C for 16 h. The first step of purification involved 6XHis-tag Ni^2+^–NTA affinity chromatography followed by gel-filtration chromatography under a buffer consisting of: 50 mM NaH_2_PO_4_, pH 6, 0.025 mM EDTA. This buffer was used in CD spectroscopy, NMR spectroscopy, and ITC experiments. Uniformly ^15^N labeled proteins were expressed in M9 media using ^15^NH_4_Cl as sole source of nitrogen.

Palindromic 12mer Dickerson-dodecamer DNA (5’-CGCGAATTCGCG-3’) sequence (DD12 dsDNA), used in this study, was commercially synthesized (Eurofin Inc). DNA was dissolved in water and solution was heated for 4 min at 95°C and annealed using snap cooling on ice (4°C) to generate a dsDNA solution.

### CD and NMR spectroscopy

CD spectra for WT and V1067G proteins were recorded on a JASCO715 spectropolarimeter at 25°C between 190nm-260nm wavelength. Thermal melting of proteins using CD was performed by thermal scans in which the protein samples (10 μM) were heated from 10°C to 90°C (at from 1°C/min rate controlled by a JASCO programmable Peltier element) and the CD signal was recorded at 222 nm. NMR spectra were recorded on a BRUKER 700 MHz NMR spectrometer equipped with HCN cryoprobe. NMR data were recorded using Topspin 3.1. Datasets were processed using Topspin3.1 and analyzed using the SPARKY software (UCSF, CA, USA).

### Isothermal titration calorimetry (ITC)

Isothermal titration calorimetry (ITC) experiments were performed using a MicroCal iTC200 machine (GE) at 25°C. Proteins (ARID WT and V1067G mutant) and DD12 dsDNA were prepared in the same buffer consisting of: 50 mM NaH_2_PO_4_, pH 6, 0.025 mM EDTA. For the titrations the sample cell was filled with 20 μM of the DNA and was titrated with 200 μM of protein. 20 injections of the titrant were done at an interval of 180 seconds. Separate protein titration in buffer was performed to determine the protein heat of dilution. The heat of dilution was subtracted from the integrated heat and the data was then fit for a one site-binding model. All the parameters were kept floating during fitting. Experiments were repeated 2–3 times for the reproducibility.

### Molecular dynamic (MD) simulation of ARID in BAF250a and V1067G mutant protein

The NMR structure of ARID domain (PDB ID: 1RYU) was used as the starting structure for the WT ARID. In the same protein, residue V1067 was replaced with G *in-silico* using mutate tool of WHAT IF server [31] to obtain the V1067G mutant system. The same MD simulation protocol was applied to both WT and mutant using the latest GROMACS 4.6.5 simulation package [32–34]. GROMOS96 45a3 united-atom force field was used to model the intramolecular protein interactions and the intermolecular interactions between the protein and solvent molecules [35]. Initial energy minimization and equilibration steps, were followed by a simulation with pressure coupling at 1 atm. During this phase, the velocities were reassigned according to a Maxwell’s distribution at 300K. Finally, the production phase of MD simulation was run keeping the temperature, pressure and number of molecules of the ensemble invariant. Production phase using 0.002ps time step was continued up to 50 ns. All the subsequent analyses were performed using different programs of the GROMACS package.

### SEG-HCA of BAF250a

We used a novel bioinformatics tool SEG-HCA (segmentation based—hydrophobic core analysis) for the prediction and characterization of the BAF250a full-length sequence for folded regions/domains [22]. SEG-HCA delineates foldable domains, by recognizing regions that are enriched with high densities of hydrophobic cluster, without employing any evolutionary information. SEG-HCA analysis predicted 10 regions in BAF250a sequence longer than 25 residues to harbor hydrophobic clusters, of which four regions that are longer than or equal to 100 residues can potentially fold into globular domains.

### Modeling of BAF250_C

PFAM predicts the BAF250_C terminal domain in BAF250a that extends between 1974–2231 residues. The novel predicted region 10 (residues 1939–2282) that includes the BAF250_C was subjected to fold recognition using PHYRE2 server [36]. The top templates identified by the approach were crystal structure of armadillo (ARM) repeats domain of human adenomatous polyposis coli (APC) (PDB Id: 3NMW, chain A) and crystal structure of the myosin chaperone UNC-45 from *C*. *elegans* in complex with a Hsp70 peptide (PDB ID: 4I2W, chain A) with which the BAF250_C shows 18–20% sequence identity.

The predicted folded region was also evaluated using the Robetta server [37]. A folded ARM repeats containing model was derived using the crystal structure of the armadillo repeat domain of APC (PDB ID: 3AU3, chain A) as reference, with a confidence score of 0.3726.

### Interacting partners in the SWI/SNF chromatin remodeling complex

We looked for the β-catenin-interacting sequence motif of DxӨӨxФx2-7E (where Ө and Ф are hydrophobic and aromatic residues, respectively) in all the annotated members in SWI/SNF chromatin remodeling complex as the probable interacting partners of BAF250_C. The search was performed through Patmatdb program of EMBOSS [38] on the different members of the SWI/SNF chromatin remodeling complex.

## Results

### Hydrophobic core analysis of BAF250a reveals folded regions in its sequence

**Fig 1:**
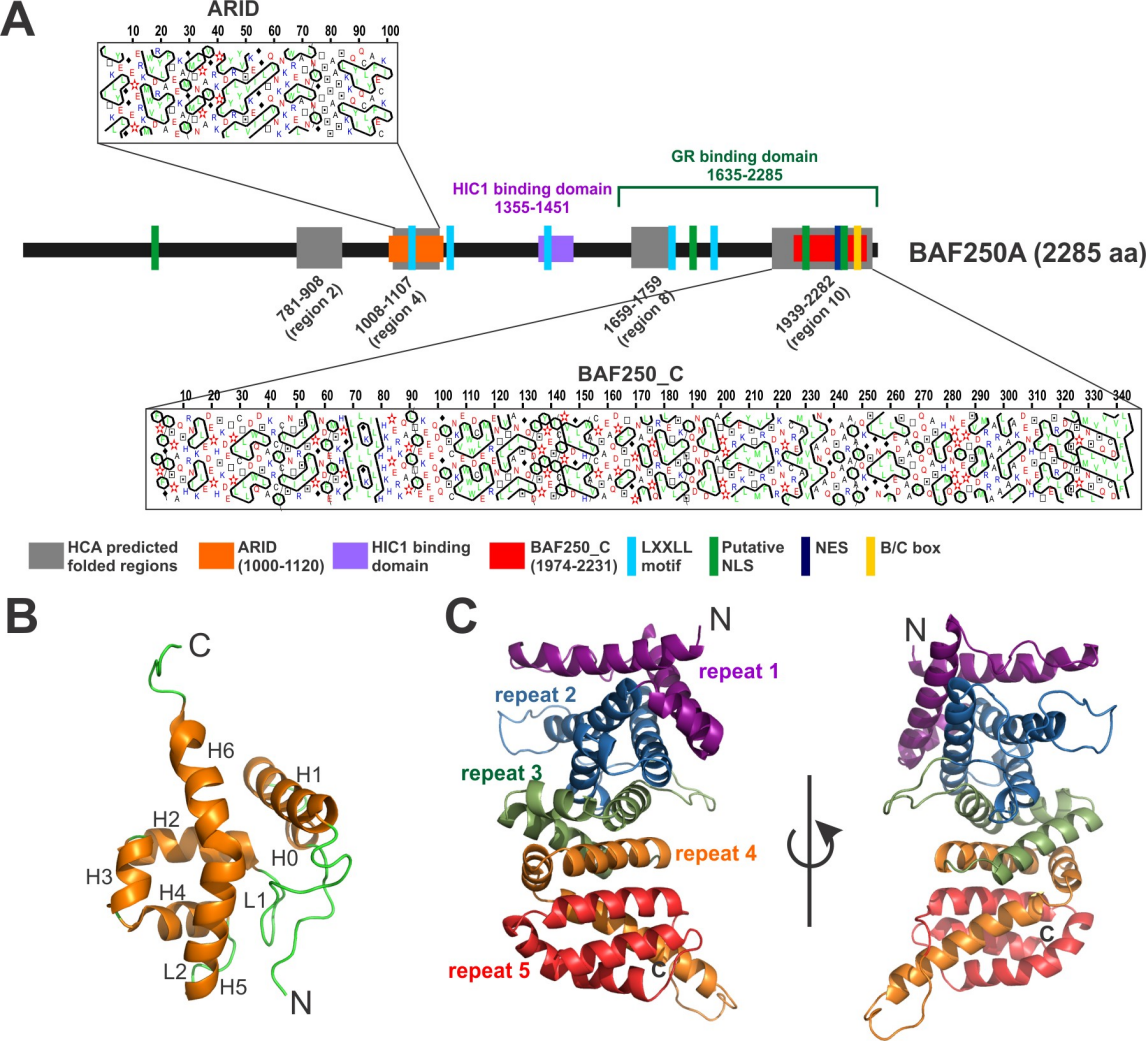
Domain architecture of BAF250a. (A) Predicted folded regions in BAF250a sequence using SEG-HCA analysis (in grey). Shown here are region 2 (781–908), region 4/ARID (1008–1107), region 8 (1659–1759) and region 10/BAF250_C (1939–2282). Previously annotated domains and motifs are marked at the bottom. (B) Previously determined structure of BAF250a ARID (PDB ID: 1RYU). Secondary structure elements are marked. (C) Robetta modeled structure of BAF250_C for the region predicted by SEG-HCA (residues 1939–2282). 5 ARM repeats in modelled BAF250_C are marked. Two views of the modelled structure are shown.

SEG-HCA (segmentation based—hydrophobic core analysis) is a novel method that delineates foldable domains using only the amino acid sequence of the corresponding protein without any evolutionary information [22]. Essentially SEG-HCA relies on the identification of regions in protein sequence with high densities in hydrophobic clusters (hydrophobic cluster analysis) [39,40]. The hydrophobic clusters mainly corresponding to regular secondary structures may finally result in folded, globular domains. We used SEG-HCA tool to predict potential folded regions in the entire length of BAF250a (S1A and S1B Fig). SEG-HCA analysis revealed several regions in BAF250a sequence that can potentially fold with a hydrophobic core. There are total 10 regions of > 25 aa predicted to have a cluster of hydrophobic residues resulting in a hydrophobic core (S1A and S1B Fig). Of these, region 2 (aa 781–908, length 128 residues), region 4 (aa 1008–1107, length 100 residues), region 8 (aa 1659–1759, length 100 residues), and region 10 (aa 1939–2282, length 344 residues) are at least 100 residues long and can potentially form independently folded domains (Fig 1A, S1A and S1B Fig). Indeed, region 4 overlaps with ARID (aa 1000–1119) and region 10 encompass PFAM annotated BAF250_C (aa 1974–2231) domains. The length of SEG-HCA predicted region 10 (residues 1939–2282) is longer than the PFAM annotated domain (residues 1974–2231) and was therefore used as a domain boundary for BAF250_C in this study. The region between these two domains has been shown to contain protein-protein interaction motifs/domains including HIC1 (*hypermethylated in cancer 1*) binding domain (aa 1355–1451) and GR (*glucocorticoid receptor*) binding domains (aa 1635–2285), which partially overlaps with BAF250_C (aa 1974–2231) [8]. Other regions at the N-terminus (aa 1–600) and regions interspersed between the ARID and BAF250_C, excluding the boundaries predicted by SEG-HCA, are rich in repetitive sequences and predicted to be disordered. However, it is possible that these intrinsically disordered regions adopt stable structure upon binding to the targets [41].

Three-dimensional structure of the N-terminal ARID of human BAF250a (SEG-HCA region 4) was solved previously using solution NMR methods (Fig 1B) [19]. However, the presumed domain in the C-terminal, reported as the longest foldable region (SEG-HCA region 10) in BAF250a, is functionally and structurally uncharacterized. Therefore, we have predicted the fold of region 10 in BAF250a using intensive PHYRE2 [36] and profile-based searches such as HHPred [42]. In both the approaches, the Armadillo (ARM) repeats were found to be associated with the fold with nearly 238 residues (93%) predicted to adopt this fold at greater than 90% accuracy. Further, we used the ab-initio approach of the Robetta webserver to model this region [37]. The server proposed a folded ARM repeats containing model using crystal structures of the armadillo repeat domain of adenomatous polyposis coli (APC) (PDB ID: 3AU3, chain A) as reference. APC is a tumor suppressor protein that regulates Wnt signaling and other cellular processes through interaction of its ARM repeats with various proteins [43]. The proposed model predicts 5 ARM repeats in the C-terminal folded domain of BAF250a (Fig 1C).

### ARIDs in different domain context cluster distinctly

**Fig 2:**
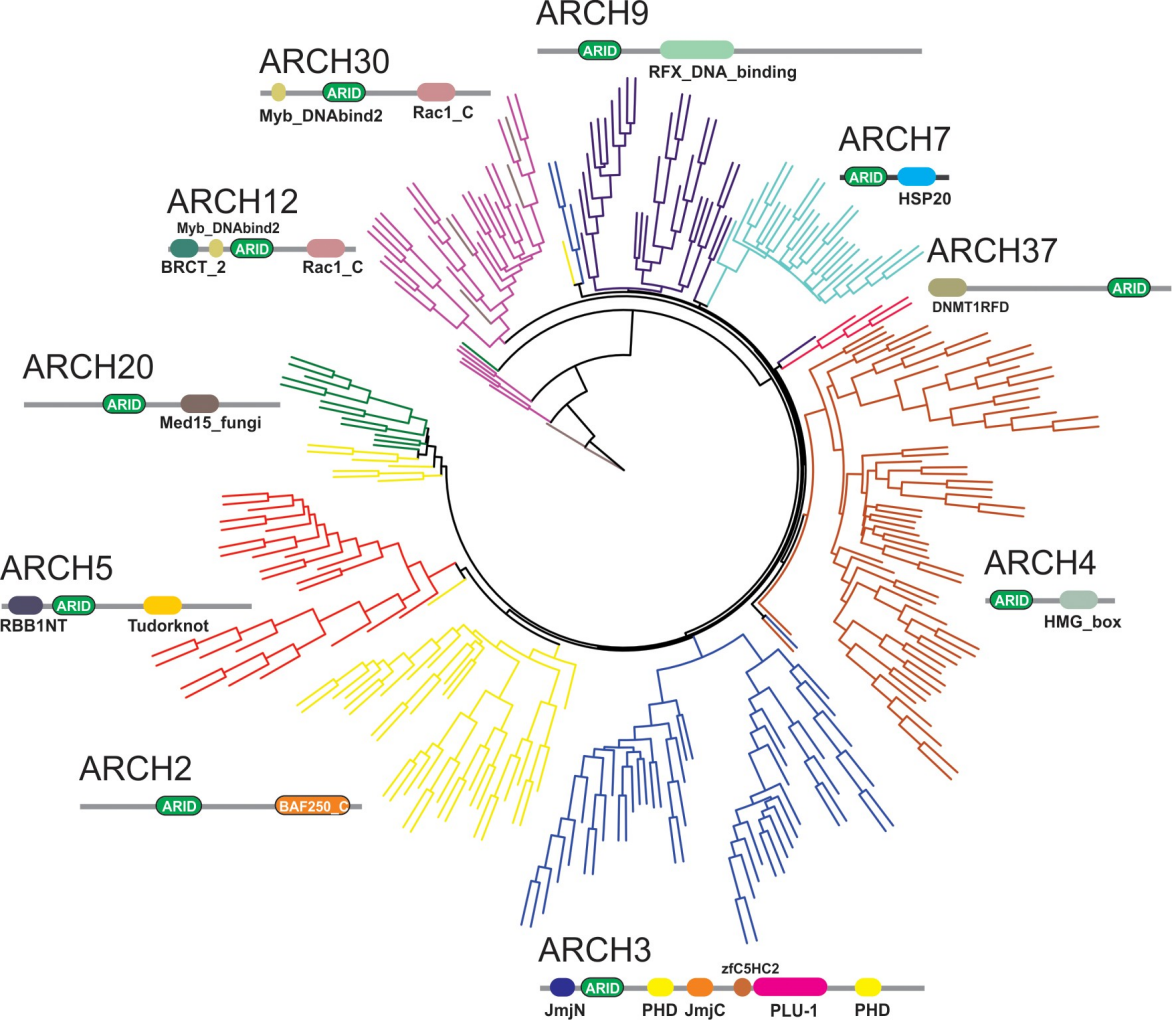
Phylogenetic tree of the ARID domain in an architecture-independent manner. Neighbor-joining tree with 1000 bootstraps of representative ARID sequences from ten different domain combinations show distinct sub-clustering for individual domain architecture, which suggests that specific sequence signatures might characterize each domain context. The corresponding domain architecture is depicted next to each sub-cluster in the tree.

Different kinds of structural and functional constraints operate on the amino acid residues in proteins during evolution. Therefore, the pattern of evolutionary constraints on amino acid positions in a protein family may differ depending on its partnering or another interacting domain in the protein [44]. Indeed, it is well appreciated that the domain contexts of a protein dictate its function, as often the same domain in different contexts results in an altogether different biological function [45]. The availability of the ARID in diverse domain contexts (as seen here in ARCH 1–11) therefore presents an opportunity to compare its sequences in each architecture. In PFAM 31.0, the ARID is found in combination with other domains in 164 domain architectures [15]. Of these, we have selected 11 major ARID containing architectures (termed ARCH1-11) (Fig 2 and S1 Table, also see Methods) to determine if the ARID in each of the architectures has a differential set of sequence signatures that can discriminate between the various domain contexts in which it occurs. Representative sequences of the ARID from each of the 10 architectures (excluding ARCH1 where it is not associated with any other domain) were aligned. The phylogenetic tree derived from this alignment through neighbor-joining and with 1000 bootstraps showed a distinct sub-clustering of the ARID sequences in an architecture-specific manner. As shown in Fig 2, this distinct sub-clustering across architectures suggests that specific sequence signals exemplify ARID in each of the diverse domain architectures that it occurs in.

### ARID and BAF250_C have co-evolved in BAF250a

BAF250a/b homologues in the SWI/SNF complexes are exclusively associated with the BAF250_C in ARCH2. Unlike ARID, which is associated with a number of other domains in various organisms; the BAF250_C is uniquely associated with the ARID (except for one instance where in addition to ARID it co-occurs with the mannosyl transferase in ARID1a of the King Cobra). Of the 236 proteins showing the BAF250_C and ARID domain combination (ARCH2), the majority of proteins are annotated as either ARID1a (BAF250a) or ARID1b (BAF250b)-like sequences or as subunits of the SWI/SNF-like ATP-dependent chromatin remodeling complexes in various eukaryotes. Some proteins are annotated as *osa* proteins while others are as yet uncharacterized proteins. Although the predicted domain contexts of both BAF250a and BAF250b proteins are similar, the two proteins share only 59% sequence identity along the entire length of the proteins. Unique association between ARID and BAF250_C domains in ARCH2 prompted us to examine the evolution of these two domains in ARCH2. For this, 210 sequences of the core ARID and those of the BAF250_C were aligned separately using MUSCLE [22]. Phylogenetic trees were generated using neighbor in the PHYLIP suite with 1000 bootstraps and visualized through iTOL [38]. The trees derived separately from the ARID and BAF250_C domains, mirror each other’s topologies (S2 Fig). This suggests that these domains are subjected to similar evolutionary pressures to co-evolve [46].

S2 Fig also shows that the trees of the core ARID and BAF250_C domains make a clear distinction between BAF250a and BAF250b-like sequences. Such a partitioning of the two paralogues and their as yet uncharacterized homologues is useful, since the tree offers clues to their proximity to BAF250a-like and BAF250b-like proteins. This partitioning could be employed in the potential annotation of a number of proteins in the dataset that are uncharacterized or unannotated. Based on this distinct partitioning, 40 sequences that were ambiguously annotated in the UniProt database were grouped into BAF250a and BAF250b like sub-clusters (S2 Fig).

### ARIDs in diverse domain contexts harbor distinct sequence fingerprints

**Fig 3:**
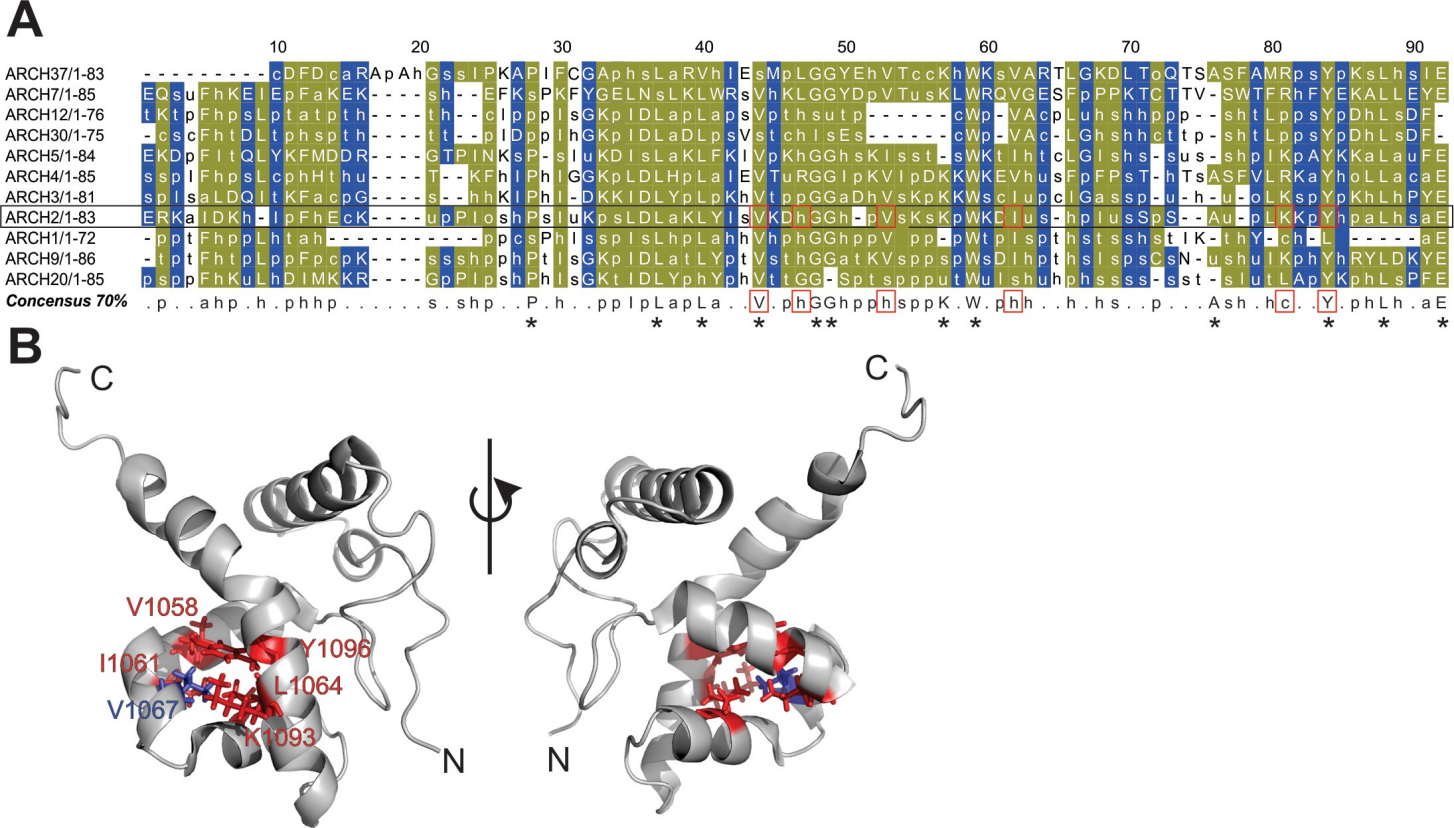
Multiple sequence alignment of consensus sequences of ARID domains across 11 different domain architectures in the dataset. (A) Each sequence in the alignment corresponds to a biochemical property-based ARID consensus signature sequence derived for the ARID from a multiple sequence alignment in each domain architecture, at 70% conservation threshold. Residue positions that are conserved in biochemical properties across the alignments are marked in olive-green and variable alignment positions in blue. At the given percentage threshold, the most discriminating equivalence class is chosen to represent the residues in the corresponding column and the associated MView symbol is displayed as follows: uppercase–standard amino acid notation; alcohol(o)—S,T; aliphatic (l)—I,L,V; aromatic(a)–F,H,W,Y; charged(c)–D,E,H,K,R; hydrophobic(h)—A,C,F,G,H,I,K,L,M,R,T,V,W,Y; negative(-)—D,E polar(p)—C, D,E,H,K,N,Q,R,S,T; positive(+)—H,K,R; small(s)—A,C,D,G,N,P,S,T,V; tiny(u)—A,G,S; turn like(t)—A,C,D,E,G,H,K,N,Q,R,S,T. Consensus signature of ARID in domain ARCH2 is shown in a black rectangle box; residue positions marked in red boxes lie in the hydrophobic core in BAF250a ARID. These positions are conserved in all ARID as shown in red boxes in the derived consensus sequence over all architectures. All other absolutely conserved positions are marked with (*) in the consensus sequence. (B) Highlighted residues in the ribbon diagram correspond to a representative sequence of known structure in Arch2. Conserved residues that constitute the hydrophobic core in ARID are mapped onto the structure of BAF250a ARID with stick representation. V1067 (in blue) lies at position 53 in the alignment. Two views of the structure are shown.

In order to determine the sequence fingerprint of the ARID in each of 11 major domain combinations with simultaneous preservation of the ARID fold, we performed sequence analysis of the ARID in each of the different domain architectures. For this, the ARID sequences within each architecture were first aligned. Representative structural templates of the ARID, where available, were included and a structure-guided multiple sequence alignment was derived using Promals3D [47]. We employed an Mview based representation to capture differences in biochemical properties of the sequences in each architecture. Indeed, a distinct consensus sequence pattern is derived for the ARID in each of the 11 architectures, suggesting that the ARID within the context of different architectures is diverse in sequence (data not shown, available on request). Subsequently, the distinct consensus signature sequences of the ARID derived from each architecture were aligned. Shown in Fig 3A, is the consensus alignment of the ARID sequence fingerprints from each architecture. The consensus alignment shows that there are a number of residue positions, which are invariant in all ARIDs in all domain contexts (residues in olive-green in Fig 3A). This suggests that these residues are likely to play a critical role in maintaining the highly conserved structure of the ARID fold across all domain combinations.

In a previous study, six residues: V1058, I1061, V1067, L1076, Y1096, and K1093 were shown to form hydrophobic core of BAF250a ARID (Fig 3B) [10]. Indeed these residue positions are well conserved in ARIDs across all domain architectures (Fig 3A, marked in red box). Other absolutely invariant residues: P1042, L1051, G1062, and W1073 are found almost identically spaced in ARID across all architectures [20]. Previously, the conserved residue Y1097 in H5, invariant residue P1042 in loop L1, and conserved residue W1073 in H4 were shown to form an aromatic structural scaffold in ARID structure [19]. A biochemical property-based conservation at each residue position of BAF250a ARID across all architectures is summarized in S2 Table.

There are residue positions where ARIDs vary from each other in an architecture-specific manner. Likely, these positions preserve the overall ARID structural fold and yet bring about specificity in each distinct architecture (residues marked in blue in Fig 3A and denoted as variable in S2 Table).

### Mutation of a residue (V1067G) of the conserved hydrophobic core leads to unfolding of BAF250a ARID

**Fig 4:**
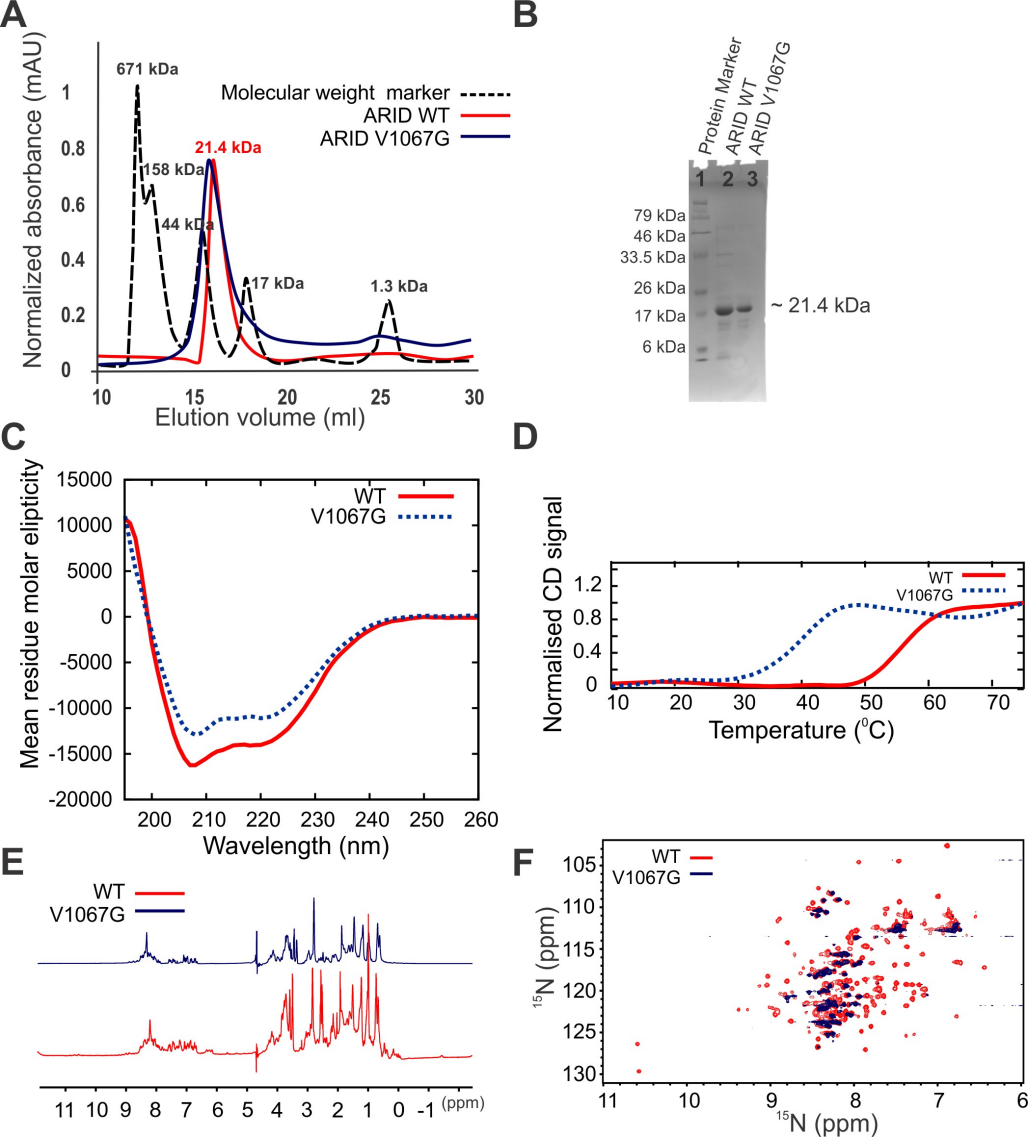
V1067G mutation destabilizes the ARID domain. (A) Elution profile of ARID WT and V1067G on size-exclusion column. (B) SDS-PAGE analysis of WT and V1067G ARID after the size-exclusion chromatography. (C) Overlay of CD spectra for WT and V1067G ARID shows loss of secondary structures upon mutation. (D) Thermal stability of WT and V1067G ARID determined by thermal denaturation using CD spectroscopy. (E) Overlay of 1D ^1^H spectra of WT and V1067G ARID. (F) Overlay of 2D ^15^N-^1^H HSQC spectra of WT and V1067G ARID.

Mutation of a residue, valine at position 1067 (V1067) to a glycine, that is part of the hydrophobic core described earlier, was shown to cause growth defects and ultimately growth arrest in mice (Fig 3A and 3B) [10]. V1067G mutation results in a protein that had reduced DNA binding affinity [19]. We expressed and purified ARID WT and V1067G mutant from *E*.*coli* BL21 (DE3) expression cells (please see Materials and Method). Both the WT and mutant protein eluted at approximate monomer size from a size-exclusion column (Fig 4A). The purity level and the identity of the proteins were ascertained using SDS-PAGE analysis and mass-spectrometry (Fig 4B and S3 Fig). We observed precipitation of V1067G mutant protein over time compared to the WT ARID that remained soluble unto 2–3 weeks.

Fig 4C shows the CD spectra of WT and V1076G mutant of ARID. The CD spectra showed that both the proteins were α-helical at 25°C, however there is loss of secondary structures in V1067G compared to the WT ARID. Further, we monitored thermal unfolding of proteins using CD spectroscopy. CD signal at 222 nm was monitored as a function of temperature from 10°C to 90°C and T_m_ of the transition was calculated. Both WT and mutant proteins showed two-state transition but with varying T_m_. While T_m_ for the WT was 56°C, the T_m_ for the V1067G mutant was 40°C showing that V1067G mutation leads to destabilization of the ARID (Fig 4D).

We also recorded 1D ^1^H and 2D ^15^N-^1^H HSQC NMR spectra of WT and V1067 mutant of ARID. The 1D ^1^H spectra clearly showed that as compared to the WT there has been gross loss of folded structure in mutant V1067G as evident from the broadened resonance peaks in the amide and methyl protons region of the spectra (Fig 4E). This was further exemplified by the inspection and comparison of 2D ^15^N-^1^H HSQC spectra of WT and V1067G mutant (Fig 4F). The cross peaks in 2D ^15^N-^1^H HSQC spectra result from every N-H correlation in protein including the backbone amide group. Therefore, the 2D ^15^N-^1^H HSQC in the amide region acts as finger print of a protein. Well-dispersed and uniform intensity cross-peaks in 2D ^15^N-^1^H HSQC spectra indicate that protein is well folded under the experimental condition. WT ARID gave a 2D ^15^N-^1^H HSQC spectra of a typically well folded protein, however the 2D ^15^N-^1^H HSQC spectra of V1067G mutant showed disappearance of well dispersed cross-peaks and appearance of overlapped and broadened resonances indicating that mutant protein is only partially folded as compared to the WT protein.

### MD simulations show that V1067A mutation results in destabilization of ARID

**Fig 5:**
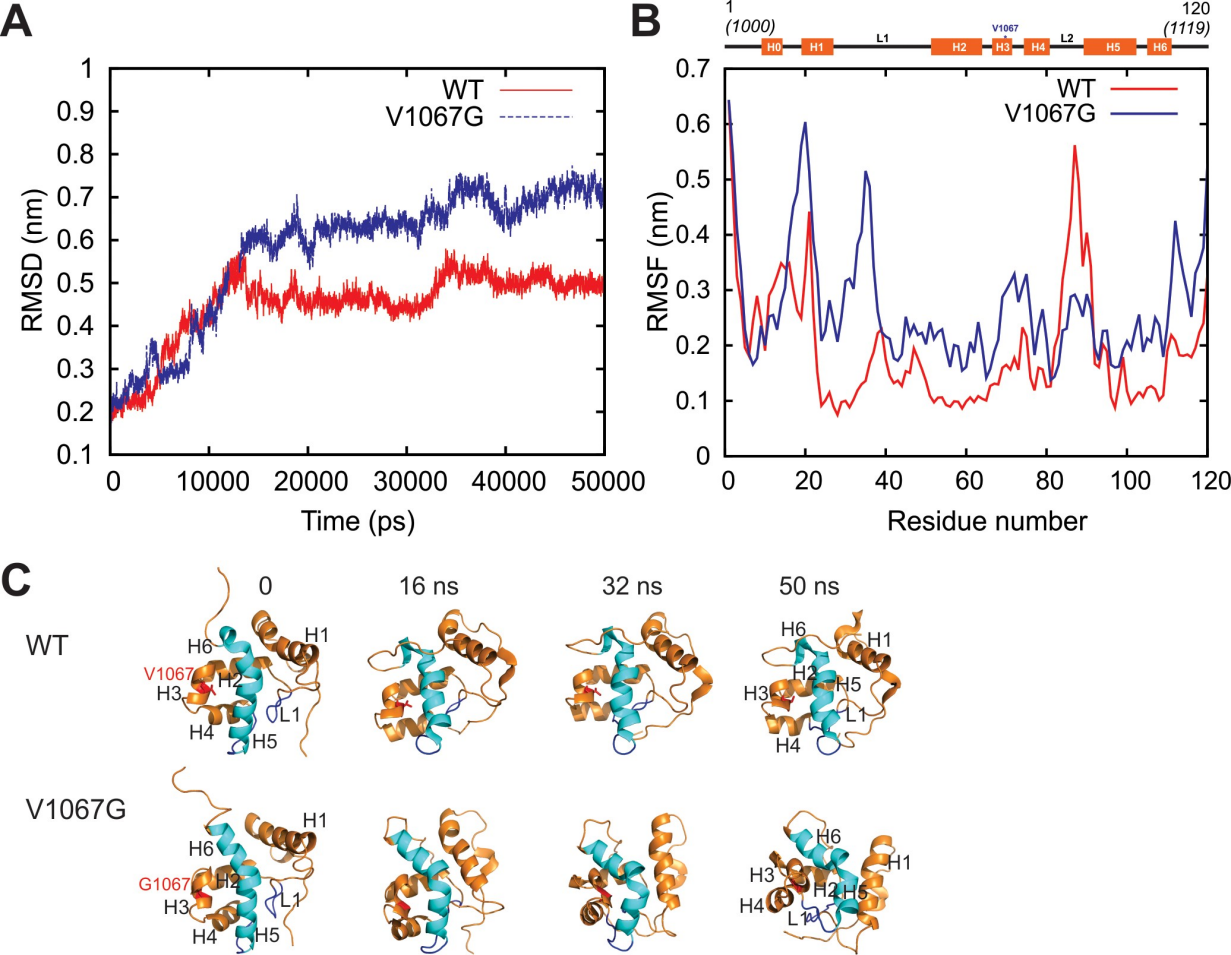
MD simulation of WT and V1067G mutant ARID. (A) RMSD plot of C-α atoms for WT and V1067G show the convergence for both the structures over 50 ns simulation time. (B) RMSF plot of C-α atoms for each residue of WT vs. V1067G are shown over 50 ns simulation time. Secondary structure of BAF250a ARID with helices as rectangular box is shown at the top. Mobility of WT increases selectively in residues near the N-terminus, loop L1, part of H4 and H5 helices compared to the mutant. (C) Snapshots of ARID structure during the course of MD simulations. Snapshots at marked time are shown. V1067 in WT and G1067 in mutant structure are shown in red. All the secondary structures are marked. Helix 5 and Helix 6 that are marked in cyan are shown to develop a knick in the mutant during simulation.

In order to gain a molecular insight into the effect of V1067G mutation on the structure of WT ARID, we performed an all-atom MD simulation of both WT (PDB ID: 1RYU) and an *in silico* generated V1067G mutant, using GROMOS96 45a3 united atom force field [35]. Comparison of Root Mean Square Deviation (RMSD) between WT and mutant over the entire simulation time exhibits less stability and higher degree of flexibility for V1067G mutant (Fig 5A). Root Mean Square Fluctuation (RMSF) over 50 ns simulation showed that the mobility of WT increases selectively in regions involved in protein-DNA interaction: residues near the N-terminus, loop L1, part of H4 and H5 helices compared to the mutant, which may contribute to DNA binding ability of WT ARID (Fig 5B). It is clearly evident that over the entire simulation time, WT protein maintained its overall structure whereas in mutant V1067G, the structure underwent distortions. Helices H5 and H6 developed a knick in mutant (Fig 5C). The structural alterations significantly affected the orientation of previously reported DNA-binding loop (L1) and helices (H4 and H5). Significant structural deviations were shown in helix H2, H3 and H4 through the 50 ns simulation time course that likely resulted in unfolding of the hydrophobic core in V1067G, which may lead to reduction in its DNA-binding affinity. Helix H1 also underwent significant positional changes in mutant over the simulation affecting the overall structure of the mutant protein.

### V1067G mutation leads to decrease in DNA binding affinity of BAF250a ARID

**Fig 6:**
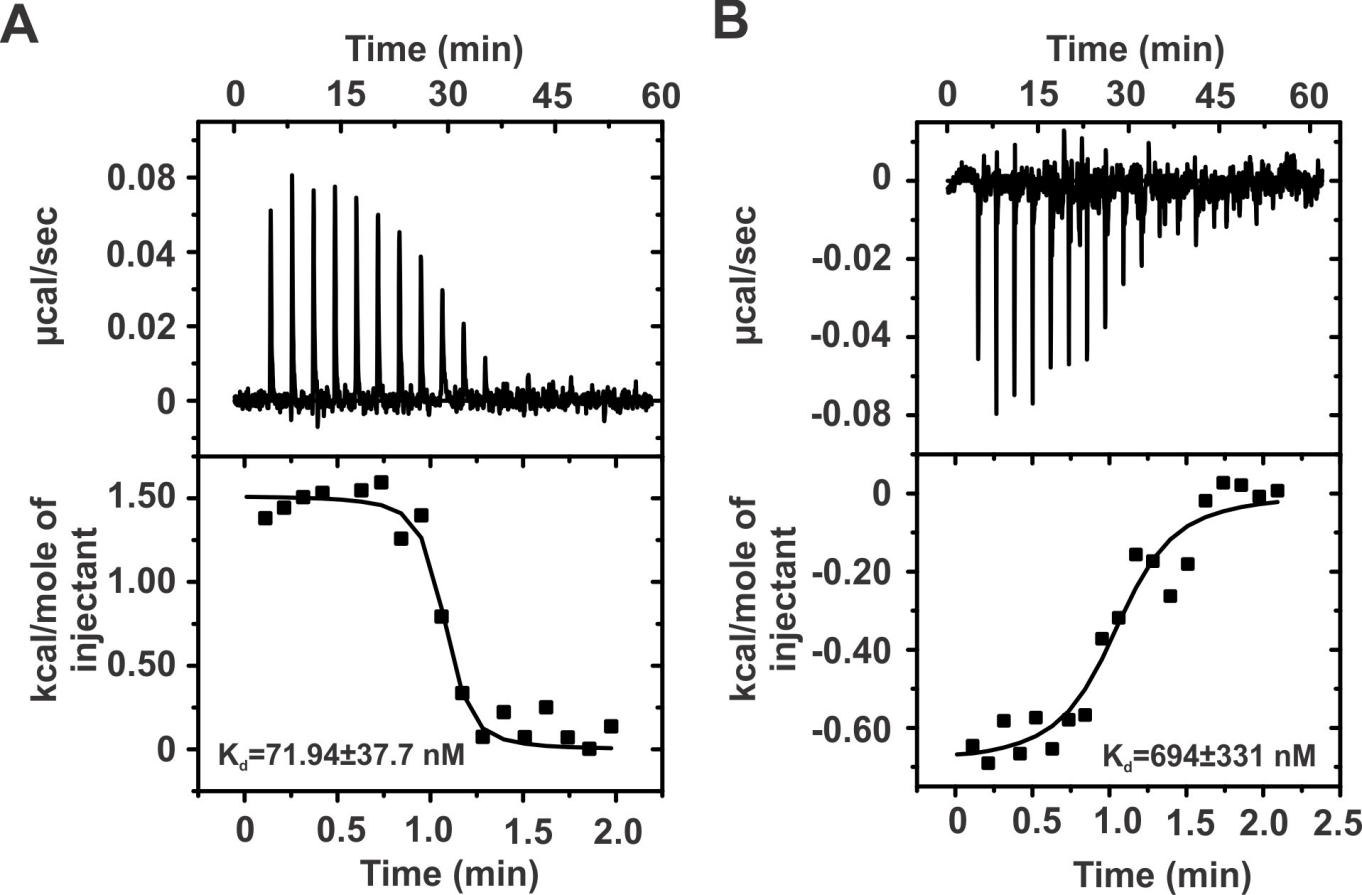
Interaction of WT and V1067G ARID with DD12 dsDNA monitored through ITC. Raw and fitted isotherms are shown and the K_d_ obtained upon fitting of the raw data is mentioned in each panel. (A) Interaction of WT ARID with DD12 dsDNA at 25°C. (B) Interaction of V1067G mutant ARID with DD12 dsDNA at 25°C.

We used isothermal titration calorimetry (ITC) to probe the interaction of WT and V1067G mutant with DNA. We used Dickerson Dodecamer sequence (DD12 dsDNA) that had been shown to interact with the ARID in a previous NMR study [19]. At 25°C, WT ARID and DD12 dsDNA complex formation proceeds with very small but a net positive enthalpy change (ΔH = 1.51±0.05 kcal/mol) with an observed stoichiometry of ~1 (Fig 6A and Table 1). Table 1 shows all the thermodynamic parameters determined from the ITC results. ARID interacts with DD12 dsDNA with K_d_ (equilibrium dissociation constant) of 71.94±37.7 nM. This is in agreement with the previous study that had reported an apparent K_d_ of ~ 50 nM for ARID and DNA interaction. It is however worth to note that a larger construct of protein that encompassed the ARID was used in that study [10].

**Table 1:** Thermodynamic parameters derived from ITC experiments for the titration of WT and V1067G ARID into the Dickerson dodecamer (DD12) dsDNA.

Experiment	K_d_ (nM)	ΔG (kcal/mol)	ΔH (kcal/mol)	TΔS (kcal/mol)	n
ARID WT–DD12 dsDNA	71.94±37.7	-9.72±5.1	1.51±0.05	11.23±5.15	1.03±0.02
ARID V1067G –DD12 dsDNA	694±331	-8.39±4.0	-0.69±0.41	7.72±4.41	1.04±0.05

In case of V1067G mutant ARID and DD12 dsDNA interaction, the complex formation proceeds with a net negative change in enthalpy (ΔH = -0.69±0.41 kcal/mol) at 25°C (Fig 6B and Table 1). The K_d_ of the interaction is 694±331 nM, which is about 10 fold higher than that for the WT ARID, clearly showing that the V1067G mutation in ARID reduces the interaction between ARID and DNA dramatically. Taken together as a proof of principle, the MD simulation and biophysical studies including CD and NMR spectroscopy, and ITC titrations showed that a change in a residue that is conserved in all the ARIDs would result in distortion of the molecular structure, and results in loss of DNA binding. A detailed thermodynamic and mutational study would be required to determine whether the reported interaction between ARID and DNA is specific or non-specific.

### A set of conserved residues that are unique for ARCH2 lie on exposed helices of BAF250a ARID involved in DNA binding

**Fig 7:**
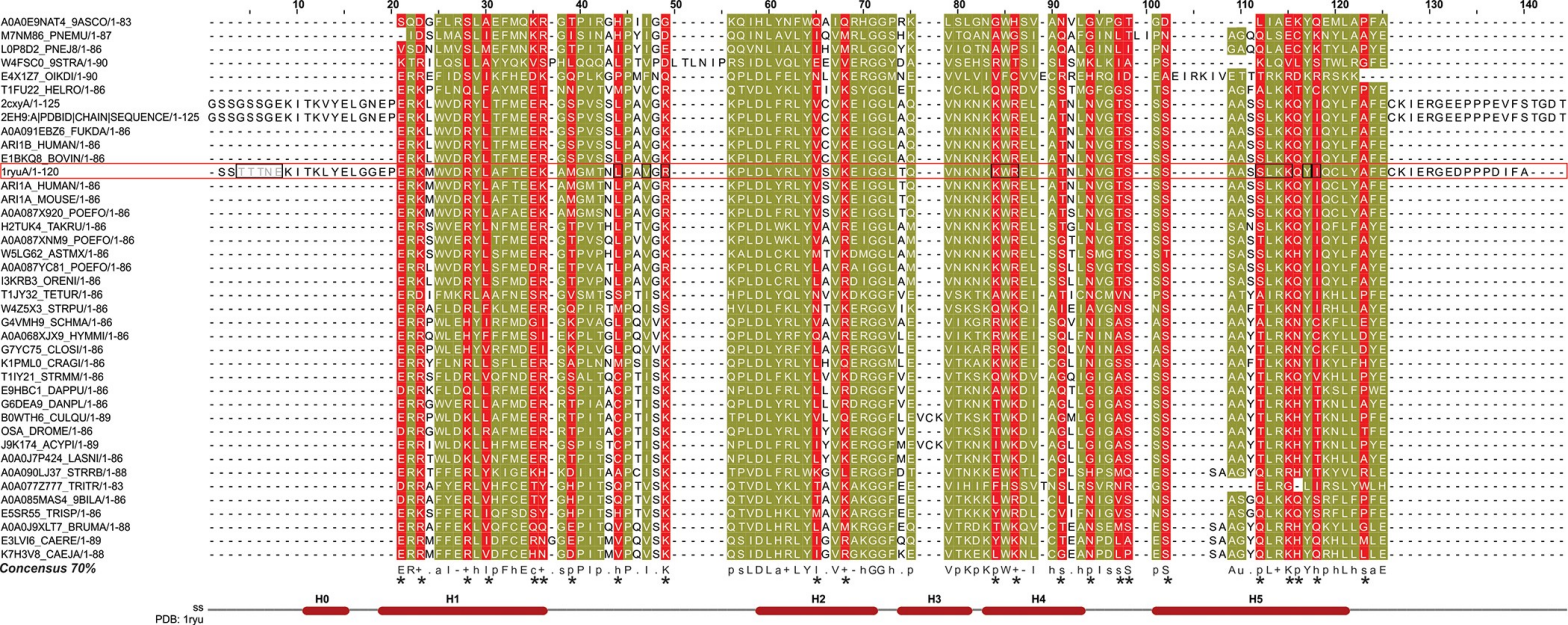
Multiple sequence alignment of ARID in ARID-BAF250_C architecture (ARCH2). The regions marked in yellow show the ARID-specific conserved signature sequence that is seen in all ARIDs across various domain architectures. Residue positions highlighted in red indicate signature residues in ARID that were selectively conserved in combination with BAF250_C. The derived biochemical property-based ARID consensus sequence for ARCH2 at 70% conservation threshold is shown below the alignment using the associated MView symbol as follows: uppercase–standard amino acid notation; alcohol(o)—S,T; aliphatic (l)—I,L,V; aromatic(a)–F,H,W,Y; charged(c)–D,E,H,K,R; hydrophobic(h)—A,C,F,G,H,I,K,L,M,R,T,V,W,Y; negative(-)—D,E polar(p)—C, D,E,H,K,N,Q,R,S,T; positive(+)—H,K,R; small(s)—A,C,D,G,N,P,S,T,V; tiny(u)—A,G,S; turn like(t)—A,C,D,E,G,H,K,N,Q,R,S,T. Secondary structure of ARCH2 representative BAF250a ARID (PDB ID: 1RYU), in a red rectangular box in the alignments) is shown at the bottom of the alignment as well. Previously reported DNA binding residues in 1RYU are marked with black box. The biochemical property-based uniquely conserved residue positions of ARCH2 in the consensus sequence are marked with (*).

To identify sequence fingerprints that distinguish ARIDs in ARCH2 from ARIDs in other architectures, we compared the consensus alignment derived for ARCH2 (Fig 7) with the consensus alignment of the ARID derived for all the other domain architectures (Fig 3A). As shown here, while a number of residue positions are conserved in all ARIDs and likely involved in formation of the core (residues in yellow in Fig 7), some positions are uniquely conserved in ARCH2 (residues in red in Fig 7).

**Fig 8:**
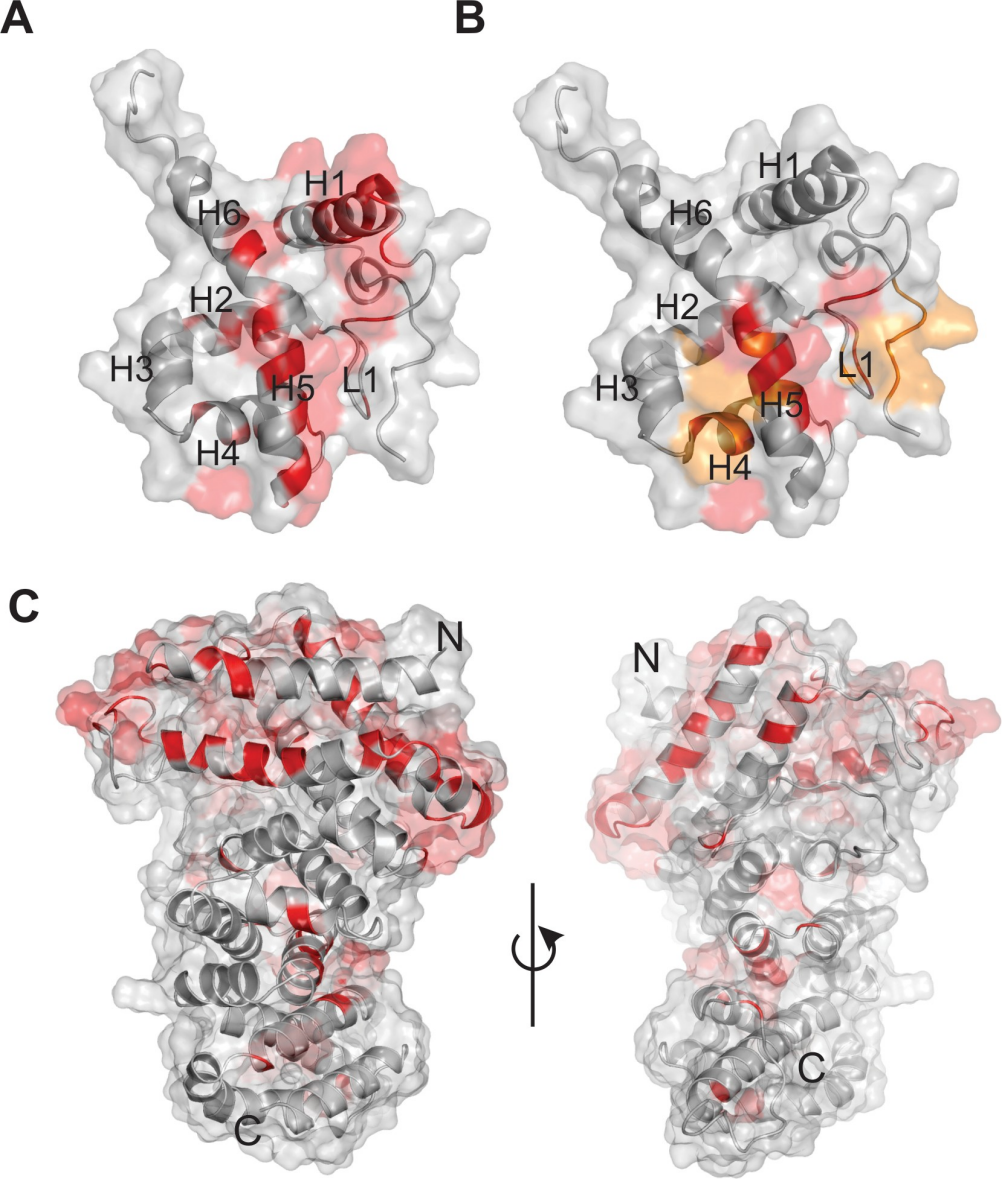
Uniquely conserved and solvent exposed residues in ARID. (A) The selectively conserved and solvent exposed residues in ARCH2 namely- E1019, K1021, R1026, L1028, E1033, K1034 in helix H1, M1036, L1041, V1044, R1046 in loop L1, V1056, K1059 in helix H2, K1072, R1074, T1078, N1081 in helix H4, T1084, S1085 in loop L2, S1087, S1091, K1094, Q1095, I1097 in helix H5 and A1102A in helix H6 are shown on the surface view of BAF250A ARID structure (PDB ID: 1RYU), in red. (B) The DNA-binding residues of ARID T1002, T1003, T1004, N1005, E1006, L1041, V1044, R1046, K1047, K1072, W1073, R1074, S1091, L1092, K1093, K1094, Y1096 and I1097 as determined in previous studies, are highlighted in yellow/red. Those DNA-binding residues that overlap with the uniquely conserved positions of the ARID in ARCH2 namely 1041L, 1046R, 1072K, 1074R, 1091S, 1094K and 1097I are colored in red. (C) The conserved and solvent exposed residues are mapped on the surface representation of the modelled BAF250_C structure (in red). Two views of the model and shown.

These residues were mapped onto the solution NMR structure of BAF250a ARID and are found distributed all over the structure (residues in red in Fig 8A). The uniquely conserved residues E1019, K1021, K1026, L1028, E1033, K1034 were found to lie on helix H1, followed by residues: M1036, L1041, V1044, R1046 in loop L1, residues: V1056, K1059 in helix H2, K1072, R1074, T1078, N1081 in helix H4, residues: T1084, S1085 in loop L2, residues: S1087, S1091, K1094, Q1095, I1097 in helix H5 and residue A1102 in helix H6. Solvent accessible surface area was calculated for these residues using Naccess [48] and several of these conserved residues were found to be surface exposed (S2 Table). This observation led us to speculate that these could be functionally important residues involved in DNA binding and/or interaction with other regions in protein including BAF250_C providing globularity to the protein.

BAF250a ARID has previously been shown to interact with DD12 dsDNA using NMR chemical shift perturbation (CSP) methods [19]. The perturbed residues are from the N-terminal region, loop L1, followed by helix H4 and H5 of the ARID and include residues: L1041, V1044, R1046, K1047, K1072, W1073, R1074, S1091, L1092, K1093, K1094, 1096Y and I1097 (red and yellow residues in Fig 8B and S2 Table). Interestingly, residues L1041, R1046, K1072, R1074, S1091, K1094 and I1097 were identified as uniquely conserved to the ARID in ARCH2 (red residues in Fig 8B and S2 Table). Therefore, we argue that the associated DNA binding functionality is likely responsible for the conservation of these surface exposed residues in BAF250a.

A number of selectively conserved and surface exposed residues found on helix H1 were not shown to be involved in DNA-binding and remain uncharacterized (Fig 8A). We speculate that these ARCH2-specific residues in ARID might be involved in potential inter-domain interactions in BAF250a or with other proteins in the BAF complex.

### Sequence and structural analysis reveal potential role of ARCH2-specific BAF250_C (in BAF250a) in mediating protein-protein interactions

A structure-based multiple sequence alignment of well-known ARM repeats containing proteins such as Adenomatous polyposis coli, p120 catenin, β-catenin and the BAF250_C domain (residues 1939–2282 of BAF250a) shows the signature three-helical Armadillo (ARM) repeats containing unit of this fold (S4 Fig and Fig 1C) (details in Materials and Methods). ARM repeats typically consist of three α-helices that are stacked in parallel resulting in a super-helical structure and are often associated with protein-binding and scaffolding functions [49,50]. The protein binding function of ARM repeat proteins involve residues from multiple repeats rather than a single repeat that has allowed this domain to diverge extensively in sequence [49,51]. The alignment also shows that the hydrophobic interactions between residues of each repeat unit, characteristic of ARM domain structures, are well conserved in BAF250_C domain (S4 Fig).

Further, we aligned the BAF250_C domains of BAF250a homologues (in ARCH2) in our dataset and found the sequences to be well conserved along the entire length (S5 Fig). High sequence conservation and solvent accessibility are two criteria that can be employed to identify surface-exposed patches of residues in a structure that are involved in protein-protein interactions. Ideally such sites should be uniformly conserved and surface exposed in all the sequences in the alignment. Therefore, we identified conserved residues in the alignment of BAF250_C homologues in our dataset (S5 Fig). Further, we computed the solvent-exposed residues in the modeled BAF250_C using Naccess (see S5 Fig legend for description) [48]. As shown here, a number of these conserved but surface exposed residues were found to lie on repeats 1 and 2 at the N-terminus, a region that was extended in BAF250_C based on our SEG-HCA analysis (Fig 8C). Therefore, we postulate that these residues are likely to function in mediating interactions between BAF250_C and other domains of the protein or with other proteins in the remodeling complex.

ARM repeat-containing proteins such as β-catenin that are known to interact with APC, cadherins and Tcf family members have been observed with asymmetric charge distribution [52]. The surface charge distribution of modelled BAF250_C is distinct from other ARM-repeat proteins such as β-catenin, APC, and plakophilin and shows the presence of a number of negatively charged residues, which include several surface exposed and conserved residues in ARCH2 (S6 Fig). Possibly these uniquely conserved and surface exposed residues in BAF250_C may facilitate the interactions of BAF250a with other proteins in the chromatin remodeling complex.

### Mapping cancer mutations of BAF250a

**Fig 9:**
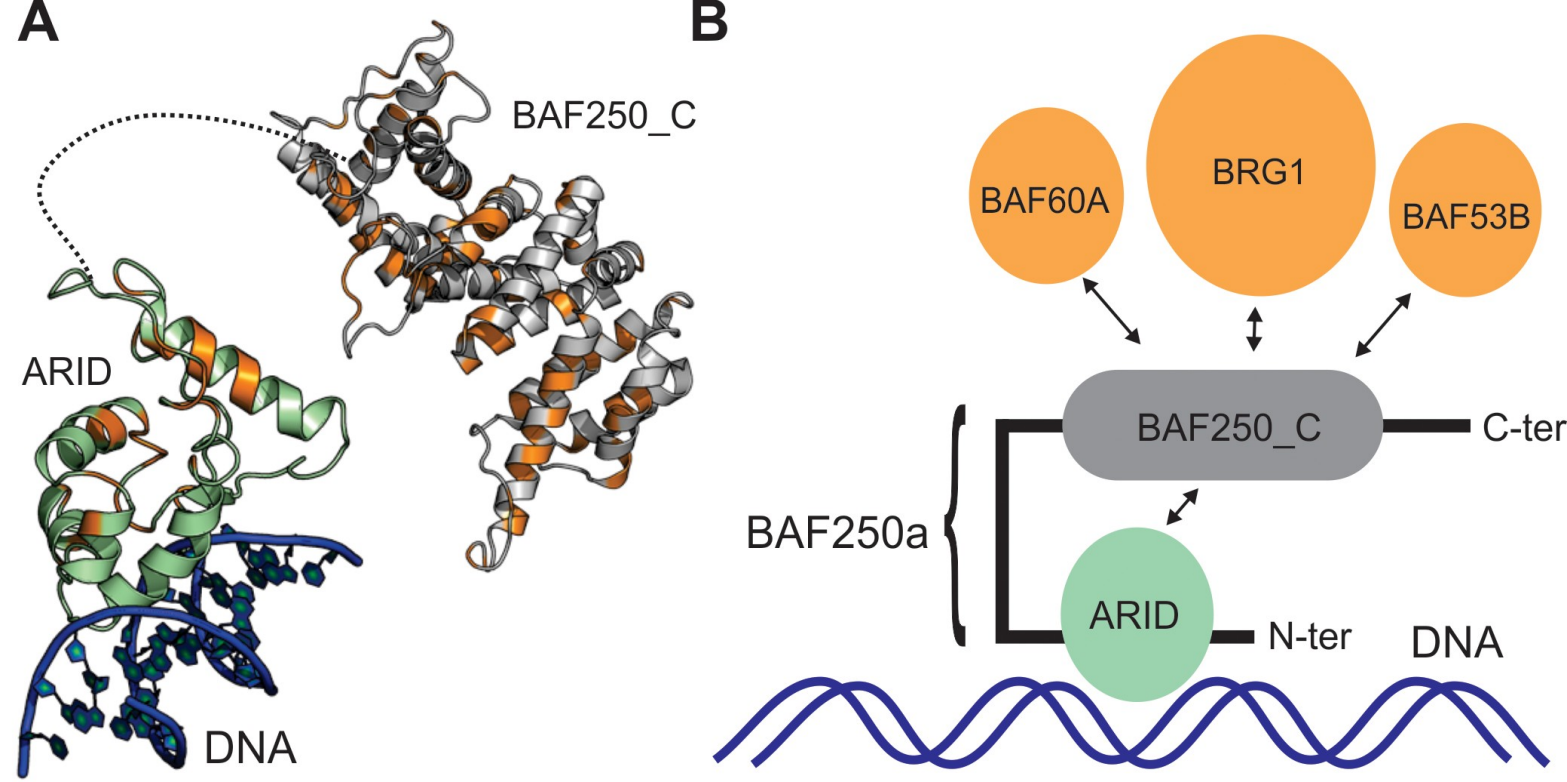
Role of BAF250a in mediating protein-DNA and protein-protein interactions. (A) Cartoon representation of BAF250_C and a plausible ARID-DNA complex are shown, where the surface exposed and conserved residues in ARID and BAF250_C which are further found to be mutated in various cancers in COSMIC database, are marked in orange. (B) Model of BAF250a interaction with DNA and other proteins. In the proposed model, the N-terminal ARID is shown to interact with dsDNA, while the C-terminal ARM-repeat BAF250_C can have inter-domain interaction with ARID and other proteins in the SWI/SNF complex.

Increasingly, evidences of the role of BAF250a in various cancers through a number of recurrent mutations of the gene have been demonstrated [1,8,13,53–55]. Since structures of the ARID and the modelled BAF250_C showed a number of uniquely conserved residues, we mapped these mutations on the structures of these two domains. The COSMIC database was searched for the most frequent somatic mutations involving the BAF250a gene distributed in 1904 unique samples [56]. COSMIC database stores the expert-curated somatic cancer mutations in proteins. Of the mutations that map to the ARID and the BAF250_C, a majority of the mutations were found to occur on surface-exposed residues (Fig 9A and S3 Table) and therefore unlikely to alter the overall structure of the individual domains but possibly alter the function of the protein. Indeed, on ARID, mutations were observed in three DNA-binding residues (T1004, R1046 and R1074). Seven observed somatic mutations involving residues V1982, R1989, W2050, L2106, G2087, L2088 and L2089 lie on the BAF250_C domain. Further, three mutations at M1022, R1026 and K1106 on the ARID occur in the residues which are conserved in ARCH2 (where ARID and BAF250_C co-occur). This raises the possibility that these mutations can potentially affect the predicted domain-domain interactions in BAF250a/b. In addition, the two LXXLL motifs on the BAF250_C domain also harbor five of the observed missense mutations, suggesting that binding sites involving interactions with nuclear hormone receptors are likely to be affected by these mutations. A large number of the missense mutations on BAF250_C are spread over the entire domain (Fig 9A and S3 Table). Indeed, a recent structure-based analysis has revealed that cancer missense mutations target protein interaction interfaces [57]. Since the ARM domain has an extensive interaction interface, different parts of which may be involved in interactions with various partnering proteins in the chromatin-remodeling complex, mutations in these different interfaces may disrupt these interactions as well.

## Discussion

Using hydrophobic core analysis based SEG-HCA approach, here we report several regions in BAF250a that harbor functionally important motifs, which are likely to be folded with a hydrophobic core. The predicted regions included the DNA binding ARID and a β-catenin like ARM-repeat fold containing BAF250_C domains. Using rigorous sequence and phylogenetic analysis, we showed that ARM-repeat fold in association with ARID is only found in BAF250a homologues (in ARCH2) suggesting that these two domains co-evolved in these proteins. While instances of the ARM repeat domains interacting with proteins that harbor a DNA-binding domain are known, their co-occurrence in a single protein is reported here for the first time [58,59]. We believe that these two domains co-occur to facilitate the interactions of the ARID containing proteins with other members of the SWI/SNF complex. Alignment of all ARID sequences (across all domain architectures) resulted in identification of a set of conserved residue positions that are critical for the maintenance of ARID fold. In a previous study a hydrophobic cluster was identified that consisted of conserved residues V1058, I1061, V1067, L1076, Y1096, and K1093. Here, we provide an experimental validation that mutation of a conserved residue (V1067G) results in gross destabilization of ARID fold that impairs its DNA binding functions. Using ITC experiments we showed that V1067G mutation leads to a drastic decrease (~10 fold) in the DNA binding affinity of ARID.

Further, alignment of ARID and BAF250_C sequence in ‘ARCH2’ resulted in identification of a specific set of conserved residues in these domains. Expectedly, this set includes residues that are important for fold maintenance but in addition, we found sets of conserved residues that are solvent exposed. This suggests that the conservation of these residues might be due to their functional importance such as DNA binding (in case of ARID) or in mediating protein-protein interactions [19].

Both BAF250a and BAF250b contain ARID and BAF250_C domain combination. The proteins differ in length with BAF250a_HUMAN being 2285 residues in length and BAF250b_HUMAN of 2236 residues length, but share 59% sequence identity along the length of the proteins. Furthermore, the sequence identity is 83% and 80% for the ARID and BAF250_C domains respectively. In spite of sharing this level of sequence identity either in full length or in folded regions, these two paralogues are functionally distinct [11,13,14]. In our sequence and phylogenetic analysis, we found that BAF250a and BAF250b sequences, though similar in domain architecture, partition into two distinct clusters. Therefore, the variable residue positions in folded ARID and BAF250_C in BAF250a and BAF250b may be functionally relevant resulting in different functional output of these proteins.

BAF250_C folds into an Armadillo (ARM) repeats fold found in other proteins such as β-catenin [22,36,42,60]. β-catenin binds to versatile partners that mediate diverse cellular functions, and essentially acts as a ‘hub’ of many cellular signalling networks. Based on the homology of BAF250_C with the β-catenin, we screened SWI/SNF complex subunits for potential BAF250_C binders. It is known from earlier studies that the ARM repeat numbered 5–9 in β-catenin forms the binding site for Tcf, cadherin, APC and ICAT. Although these β-catenin-binding partners have no apparent evolutionary relationship, they have all been shown to possess the same binding motif DxӨӨxФx2-7E (where Ө and Ф are hydrophobic and aromatic residues, respectively) to interact with positively charged lysine residues (K312 and K435 in β-catenin) by forming salt bridges [43,49,61–67]. We, therefore performed searches for this sequence motif over different members of the SWI/SNF chromatin remodeling complex that included: BCL (B-cell lymphoma/leukemia) 11A and 11B, BCL7A, BCL7B and BCL7C, BRG1 (Brahma-related gene-1), BAF (BRG1-associated factor) BAF57, BAF170, BAF155, BAF45B, BAF45C, BAF45D, BAF60A, BAF60B, BAF60C, SNF5, BRM, BAF53A, BAF53B, beta-actin, ss18, CREST and BRD9 (Bromodomain containing 9). The conserved DxӨӨxФx2-7E motif was identified in BAF60B (aa 225–235: DKVASWELRVE), BRG1 (aa 1341–1353: DELPSWIIKDDAE), and BAF53B (aa 213–224:DIIPPYMIAAKE). Therefore, we postulate that BRG1, BAF60a and BAF53b can potentially interact with BAF250a interacting partners *via* their association with BAF250_C (Fig 9B). Structural superimposition of our Robetta modeled structure of BAF250_C and the β-catenin (PDB ID: 2BCT) revealed two positively charged lysine residues, K41 in 1^st^ repeat and K186 in 3^rd^ repeat of ARM-fold in BAF250_C which are spatially equivalent to K312 and K435 of β-catenin. The two lysine residues are ~30 Å apart in BAF250_C structural model which is comparable to the measured distance of ~32 Å in the crystal structure of β-catenin (PDB ID: 2BCT) (52). Therefore, we speculate that these lysine residues in BAF250_C can potentially form salt bridges with aspartic acid and glutamic acid residues in the DxӨӨxФx2-7E motif of the predicted binding partners.

In order to test the direct binding of BAF250_C with the putative binding partners, we made attempts to express and purify the recombinant BAF250_C from E.coli. However, it expressed in insufficient quantities (in soluble fraction of *E*.*coli* lysate) hampering our attempts to purify the protein (S1 Text and S7 Fig). When interaction databases such as BIOGRID and IntAct were searched for the interactors of BAF250a we find that BRG1 is listed as a top interactor, and BAF60A as potential interactors of BAF250a [68,69] The interaction of BRG1 with BAF250a has been demonstrated elsewhere as well [18,70]. In a recent molecular modelling study, the C-terminal region of BAF250a was predicted to interact with BAF60A [71], although more experiments are needed to verify this. BAF250b was also shown to repress Wnt/β-catenin signaling through its interaction with BRG1 in a separate study. The BRG1 interacting residues were mapped to the C-terminal BAF250_C region of the protein in this study [72]. It has been suggested that the C-terminal BAF250_C containing region has putative functional motifs such as nuclear export signals (NES) and LXXLL-motif that are likely involved in mediating protein-protein interactions in several proteins including nuclear hormone receptor such as glucocorticoid receptor (GR) [71,73]. BAF250b has also been shown to acts as an E3 ubiquitin ligase that targets histone H2B at lysine 120 [74]. Thus far the ubiquitin ligase activity has not been directly shown for BAF250a, however this activity in BAF250b was mapped to the ARM-repeat containing BAF250_C that is present in both BAF250a and BAF250b. All these observations suggest that the ARM repeat containing BAF250_C in BAF250a/b is involved in multiple protein-protein interactions, both within the SWI/SNF complex as well as with other proteins (Fig 9B). Indeed, several of the observed somatic mutations in various cancers map to the large surface-exposed regions of both the ARM and ARID domains, likely interrupting such interactions (Fig 9A).

In conclusion, our analysis predicts that the BAF250a with its N-terminal ARID that binds DNA and ARM repeat BAF250_C that mediates its interactions with other proteins can recruit the human BAF complex to its targets through protein-DNA and protein-protein interactions (Fig 9B) [75]. Future experiments targeting these domains will help validate the predicted interactions of ARID and ARM repeat containing BAF250_C and reveal the function of BAF250a in the BAF complex.

## Supporting information

S1 Fig SEG-HCA based prediction of folded regions in the full length BAF250a sequence. (A) Predicted regions longer than 25 amino acids (regions 1–10) are marked with a box. (B) Schematic representation of HCA predicted regions on the BAF250a.
(TIF)

S2 Fig Phylogenetic analysis of the ARID and BAF250_C domains in ARCH2. Neighbor-joining tree for individual alignments of ARID and BAF250_C was inferred using the PHYLIP package and drawn using the iTOL server (see text for details) for (A) ARID and (B) BAF250_C. The two trees share similar topology with three main clusters as BAF250a, BAF250b and Trithorax group protein OSA, that are represented in orange, green and blue, respectively. Black lines point to entries that are currently uncharacterized and neither associated with BAF250a or BAF250b in UniProt.
(TIF)

S3 Fig **MALDI-MS spectra of purified WT (upper panel) and V1067G ARID (lower panel).** The expected masses are written in each panel and the mass found after MS analysis is encircled.
(TIF)

S4 Fig Structure-guided multiple sequence alignment of the BAF250_C of BAF250a with top templates predicted by various methods. All the templates adopt the ARM-repeat fold. Alignments were generated with Promals3D and visualized using Espript. Boxed regions are conserved and primarily hydrophobic residues that are characteristic of the Arm-repeat proteins.
(TIF)

S5 Fig Multiple sequence alignment and sequence conservation of BAF250_C in ARCH2. (A) Multiple sequence alignment is shown for homologous BAF250_C domains (1939–2282), expanded at both ends based on the region suggested by the SEG-HCA analysis (Fig 1). Only the seed sequences for this domain in PFAM are shown here. Solvent exposed as well as conserved residues across all sequences are highlighted at 70% conservation threshold and were derived by consulting the alignment of 236 BAF250_C homologues (S2 Fig). The symbols used in the consensus are as follows: uppercase–identity; lowercase–consensus level > 0.5; !–I,V; $—L, M; %—F,Y; #—NDQEBZ. Solvent accessibility is shown at the bottom of the alignment along a bar with blue for solvent exposed residues, cyan for partially exposed residues and white for solvent inaccessible residues.
(TIF)

S6 Fig Surface charge properties of ARM repeat domain in various proteins. (A) BAF250_C modelled in this study, shows predominantly negatively charged patches that is also uniquely conserved in homologs of BAF250_C (Fig 9A). (B) β-catenin, is known to bind multiple partners along the positively charged groove running through the entire surface. (C) In Adenomatous polyposis coli protein (APC), the positive groove might be the recognition site for APC-binding partners. The acidic patch is used to interact with β-catenin. (D) Plakophilin shows a predominantly positively charged groove. Blue represents regions of positive potential and red regions of negative potential, at the 10 kT/e level.
(TIF)

S7 Fig Expression and purification attempts of BAF250_C. (A) Expression test analysis of BAF250_C in *E*.*coli*. At 26°C and 37°C we observed specific induction of BAF250_C protein (marked with a red arrow). (B) Purification of BAF250_C using 6XHis-tag Ni^2+^–NTA affinity chromatography. No significant enrichment and purification of protein was achieved after affinity chromatography. (C) Western-blot analysis of different fractions after Ni^2+^–NTA column, probed using anti His-tag antibodies. The Western-blot analysis showed presence of BAF250_C in the elution fractions, however the protein showed degradations.
(TIF)

S1 Table Details of the unique set of domain combinations of the ARID to derive domain-context specific sequence signatures of the ARID domain. 11 major ARID containing architectures are shown in the table.
(DOCX)

S2 Table Biochemical property-based conservation at 70% level for each residue in all ARIDs across 11 different domain architectures *vs*. ARID in ARCH2 (BAF250a ARID, PDB ID: 1RYU). If particular residue is found to be conserved, single letter amino acid code is written in bracket next to the biochemical nature of the amino acids. * Marked indicate the position that is selectively conserved in ARID in ARCH2 as compared to all other architectures. DNA binding residues from previous study (reference # 19 in the main text) were highlighted in bold face. Therefore, functionality of the uniquely conserved residues (*) may be attributed as DNA binding where it is written in bold.
(DOCX)

S3 Table List of missense mutations reported for the ARID1A in the COSMIC database. Only mutations that map to the ARID and BAF250_C terminal domains are listed here.
(DOCX)

S1 Text Details of cloning, expression, and purification attempts on BAF250_C. (DOCX)

## Abbreviations

ARID: AT rich interacting domain
SWI/SNF: SWItch/Sucrose Non-Fermentable
NMR: nuclear magnetic resonance
HADDOCK: High Ambiguity Driven protein-protein DOCKing
CD: circular dichroism
