# Supplementary figures and images for: Domain architecture of BAF250a reveals the ARID and ARM-repeat domains with implication in function and assembly of the BAF remodeling complex
Source: PLoS One. 2018 Oct 11;13(10):e0205267. doi: 10.1371/journal.pone.0205267 (PMC6181354; doi:10.1371/journal.pone.0205267)

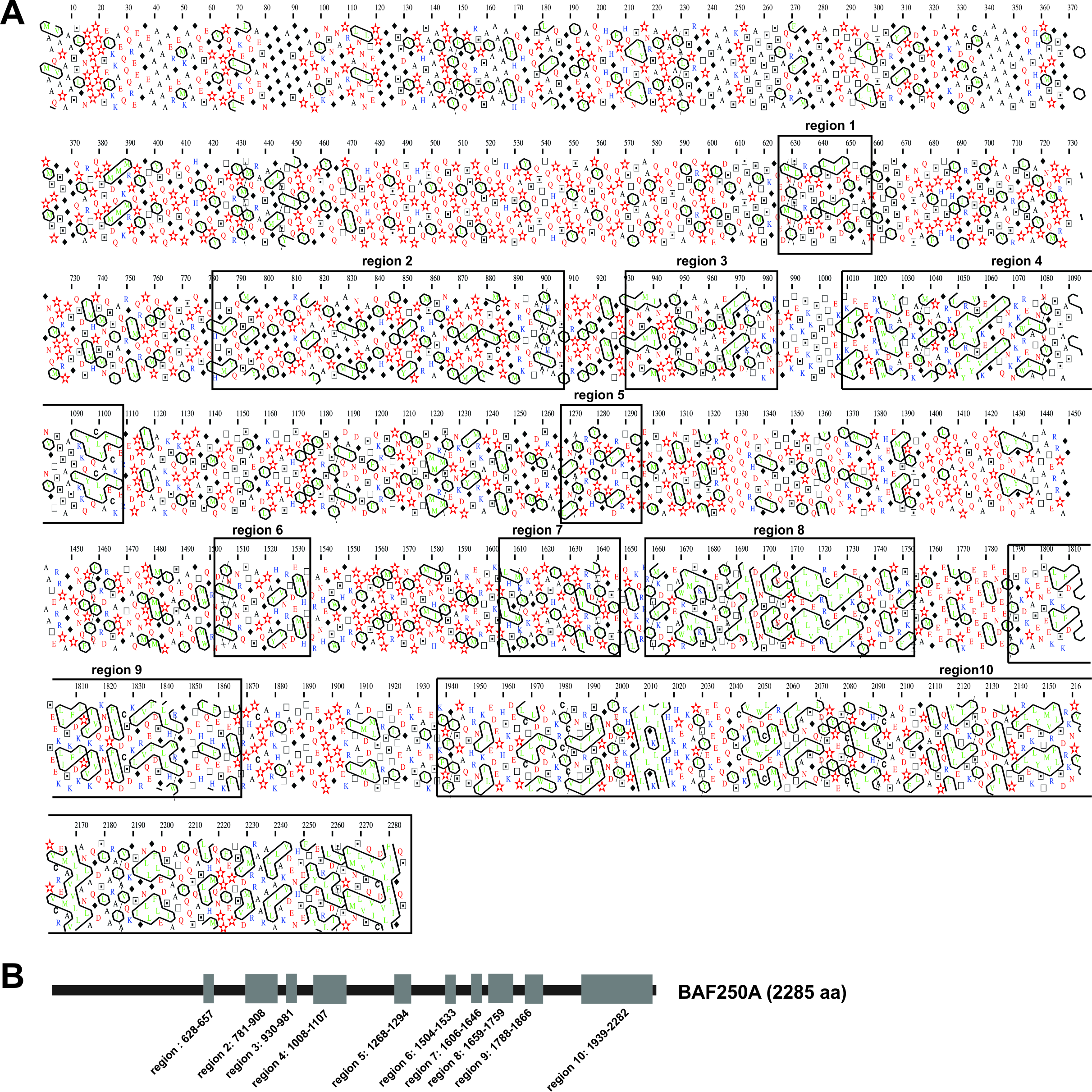

Supplement: S1 Fig — (A) Predicted regions longer than 25 amino acids (regions 1–10) are marked with a box. (B) Schematic representation of HCA predicted regions on the BAF250a. (TIF) [file pone.0205267.s001.tif]

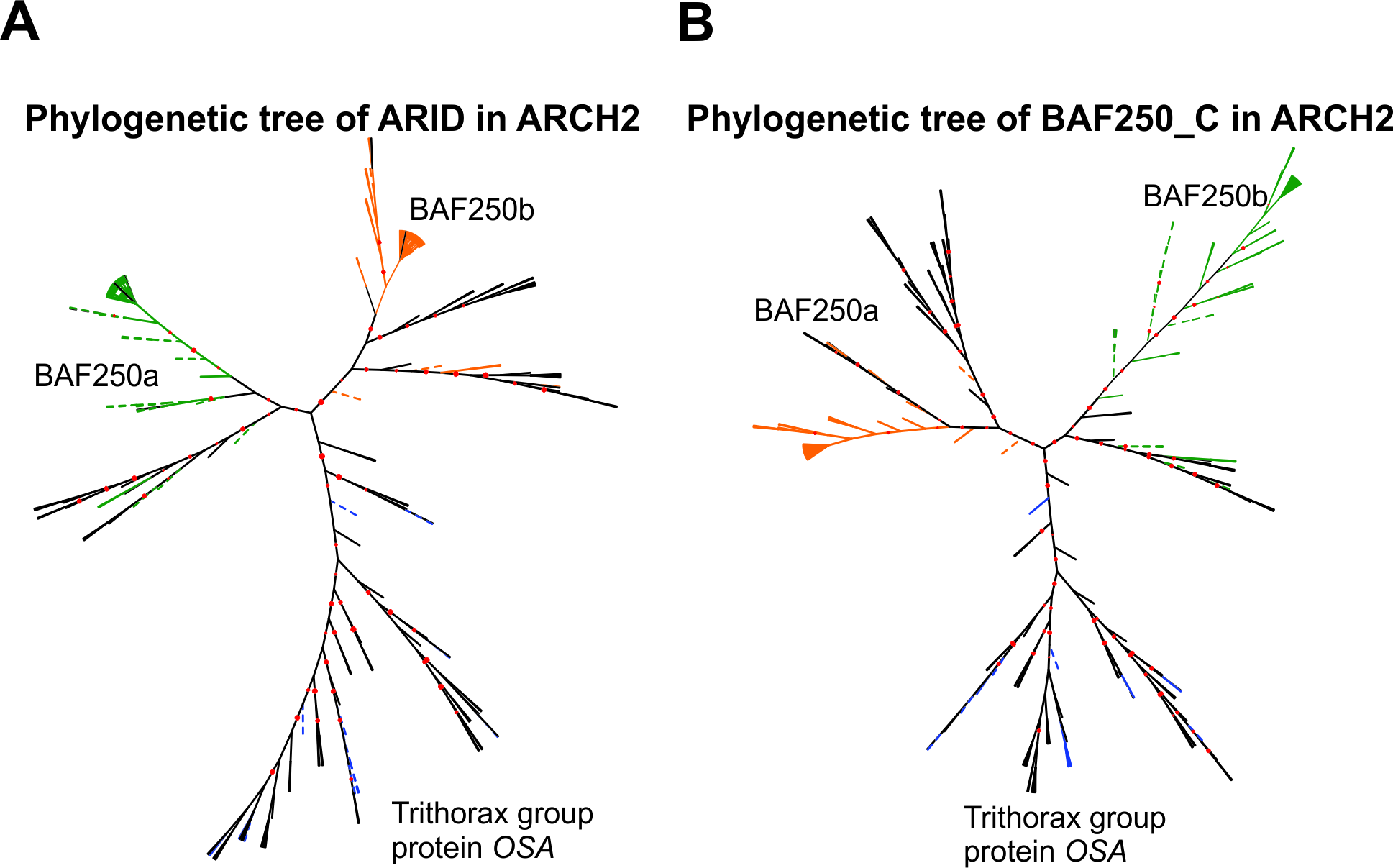

Supplement: S2 Fig — Neighbor-joining tree for individual alignments of ARID and BAF250_C was inferred using the PHYLIP package and drawn using the iTOL server (see text for details) for (A) ARID and (B) BAF250_C. The two trees share similar topology with three main clusters as BAF250a, BAF250b and Trithorax group protein OSA, that are represented in orange, green and blue, respectively. Black lines point to entries that are currently uncharacterized and neither associated with BAF250a or BAF250b in UniProt. (TIF) [file pone.0205267.s002.tif]

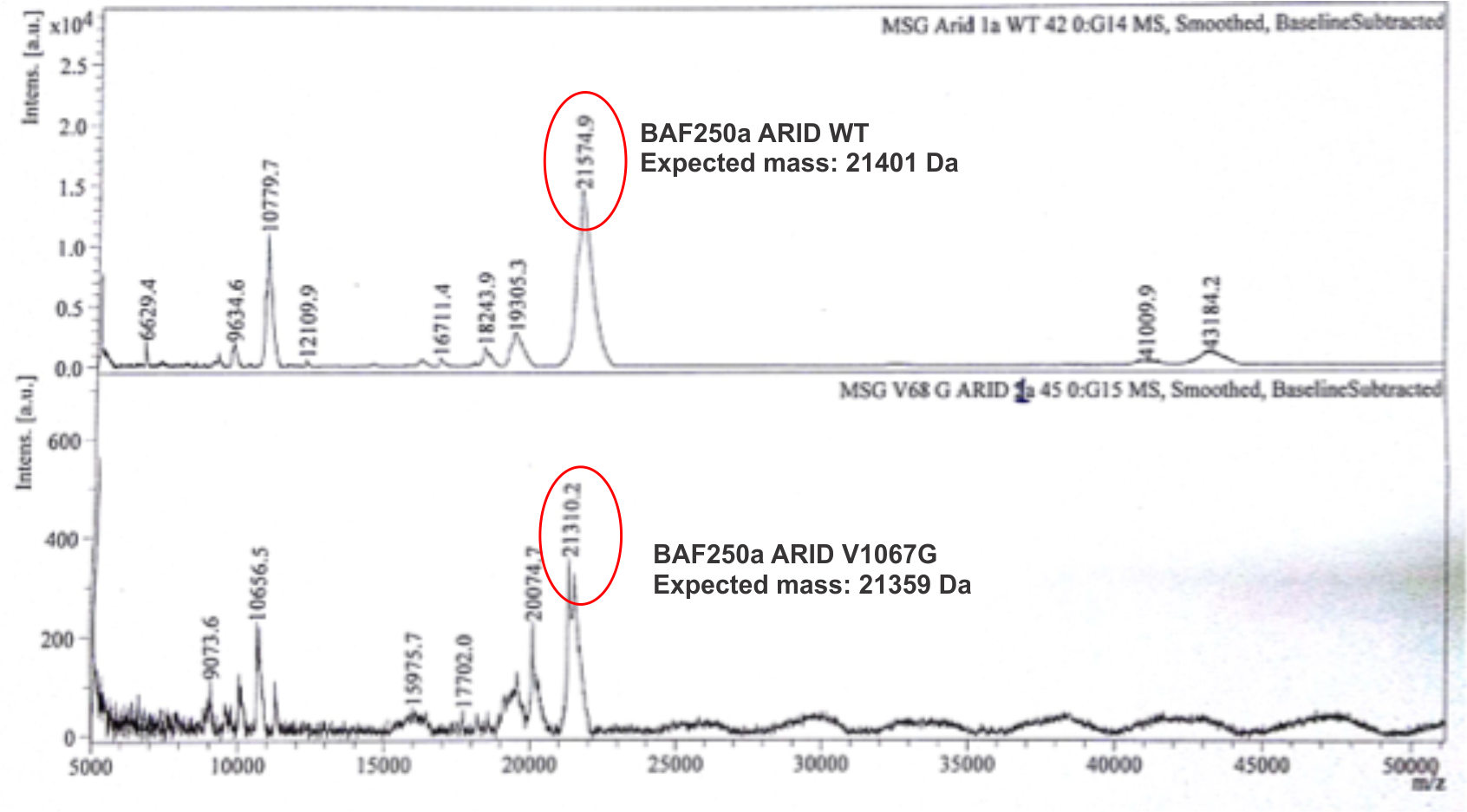

Supplement: S3 Fig — MALDI-MS spectra of purified WT (upper panel) and V1067G ARID (lower panel). The expected masses are written in each panel and the mass found after MS analysis is encircled. (TIF) [file pone.0205267.s003.tif]

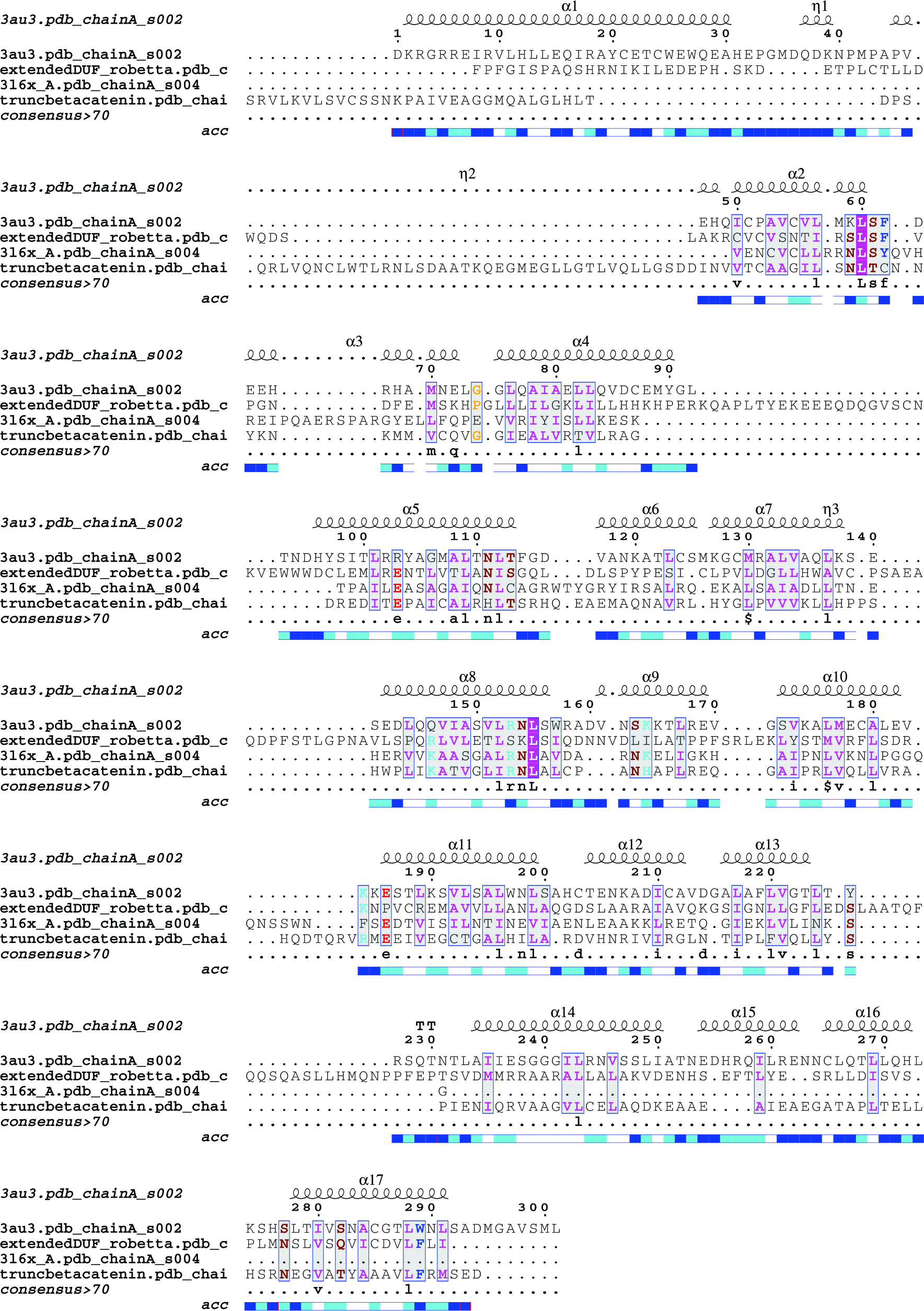

Supplement: S4 Fig — All the templates adopt the ARM-repeat fold. Alignments were generated with Promals3D and visualized using Espript. Boxed regions are conserved and primarily hydrophobic residues that are characteristic of the Arm-repeat proteins. (TIF) [file pone.0205267.s004.tif]

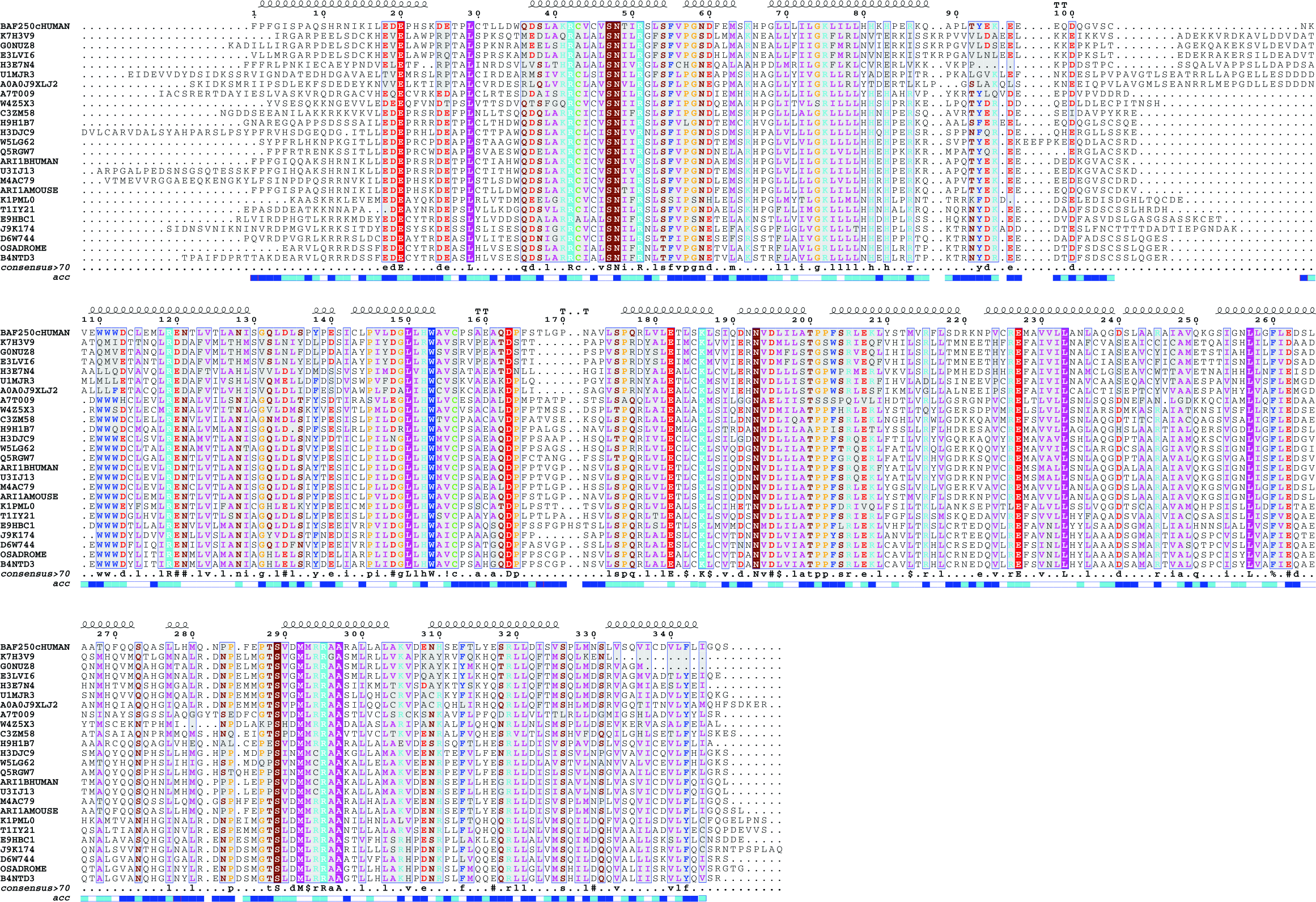

Supplement: S5 Fig — (A) Multiple sequence alignment is shown for homologous BAF250_C domains (1939–2282), expanded at both ends based on the region suggested by the SEG-HCA analysis (Fig 1). Only the seed sequences for this domain in PFAM are shown here. Solvent exposed as well as conserved residues across all sequences are highlighted at 70% conservation threshold and were derived by consulting the alignment of 236 BAF250_C homologues (S2 Fig). The symbols used in the consensus are as follows: uppercase–identity; lowercase–consensus level > 0.5; !–I,V; $—L, M; %—F,Y; #—NDQEBZ. Solvent accessibility is shown at the bottom of the alignment along a bar with blue for solvent exposed residues, cyan for partially exposed residues and white for solvent inaccessible residues. (TIF) [file pone.0205267.s005.tif]

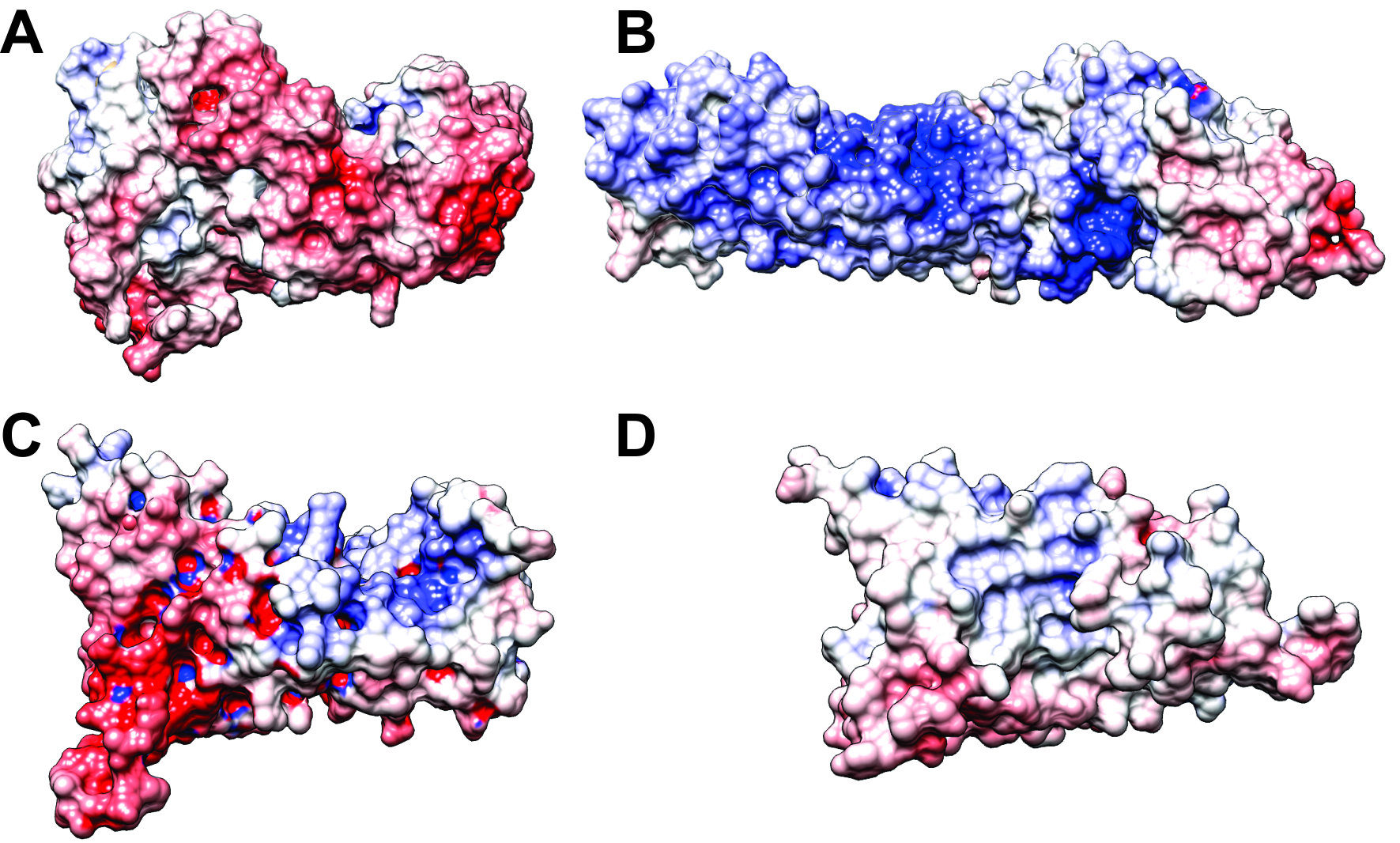

Supplement: S6 Fig — (A) BAF250_C modelled in this study, shows predominantly negatively charged patches that is also uniquely conserved in homologs of BAF250_C (Fig 9A). (B) β-catenin, is known to bind multiple partners along the positively charged groove running through the entire surface. (C) In Adenomatous polyposis coli protein (APC), the positive groove might be the recognition site for APC-binding partners. The acidic patch is used to interact with β-catenin. (D) Plakophilin shows a predominantly positively charged groove. Blue represents regions of positive potential and red regions of negative potential, at the 10 kT/e level. (TIF) [file pone.0205267.s006.tif]

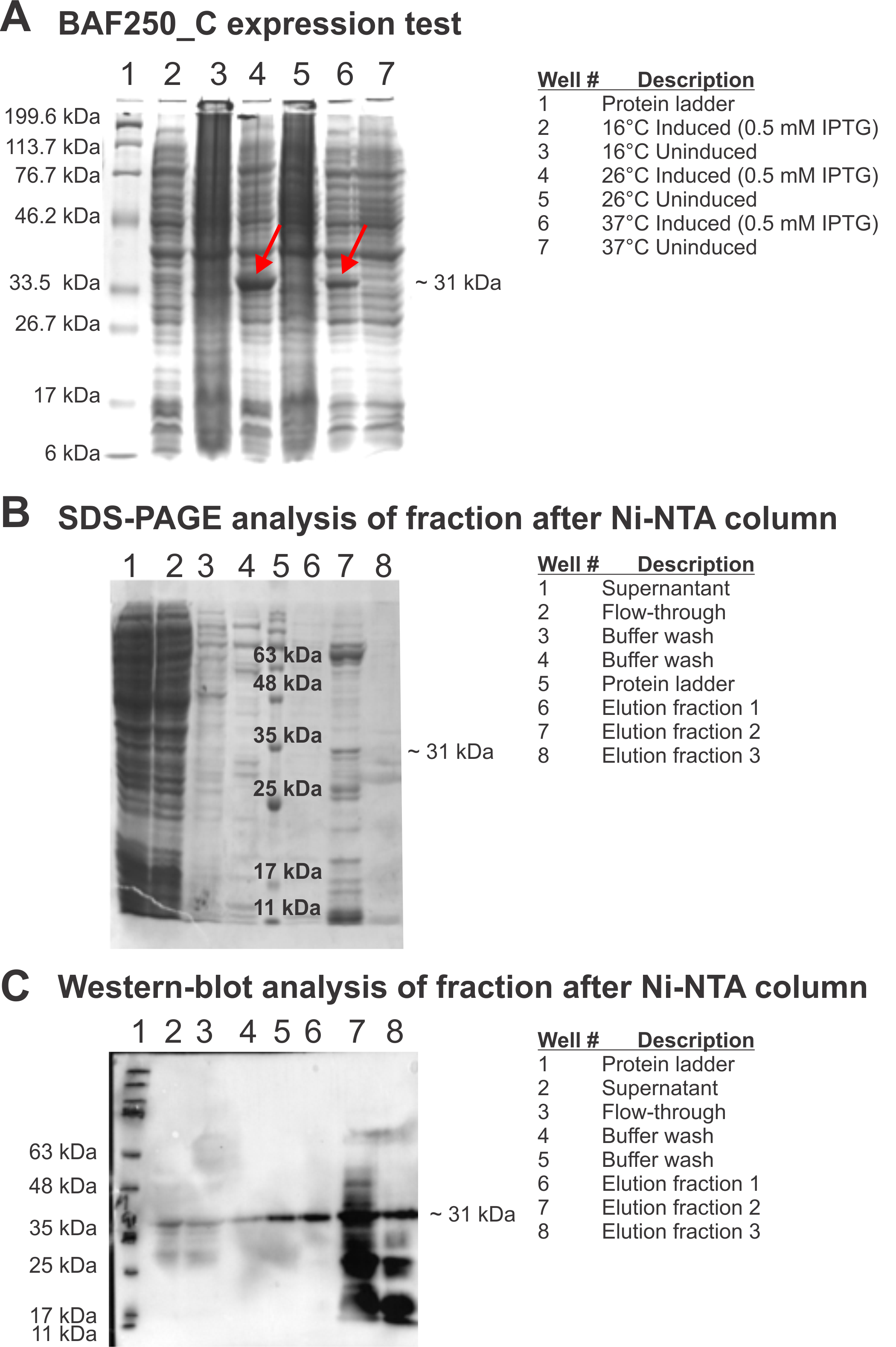

Supplement: S7 Fig — (A) Expression test analysis of BAF250_C in E.coli. At 26°C and 37°C we observed specific induction of BAF250_C protein (marked with a red arrow). (B) Purification of BAF250_C using 6XHis-tag Ni2+–NTA affinity chromatography. No significant enrichment and purification of protein was achieved after affinity chromatography. (C) Western-blot analysis of different fractions after Ni2+–NTA column, probed using anti His-tag antibodies. The Western-blot analysis showed presence of BAF250_C in the elution fractions, however the protein showed degradations. (TIF) [file pone.0205267.s007.tif]
