# Supplementary material for: Domain architecture of BAF250a reveals the ARID and ARM-repeat domains with implication in function and assembly of the BAF remodeling complex
Source: PLoS One. 2018 Oct 11;13(10):e0205267. doi: 10.1371/journal.pone.0205267 (PMC6181354; doi:10.1371/journal.pone.0205267)
Supplement: S1 Table — 11 major ARID containing architectures are shown in the table. (DOCX) [file pone.0205267.s008.docx]

**S1 Table. Details of the unique set of domain combinations of the ARID to derive domain-context specific sequence signatures of the ARID domain.** 11 major ARID containing architectures are shown in the table.

| **S.No** | **Architecture** | **Domain combinations** | **Total number of sequences** | **Number of sequences after clustering** | **Average length of the ARID domain** | **Number of sequences that were finally considered** |
| --- | --- | --- | --- | --- | --- | --- |
| 1 | ARCH1 | ARID | 1395 | 595 | 87 | 531 |
| 2 | ARCH2 | ARID, BAF250_C | 239 | 44 | 87 | 37 |
| 3 | ARCH3 | JmjN,ARID,PHD,JmjC,  zfC5HC2,PLU1,PHD | 234 | 51 | 85 | 47 |
| 4 | ARCH4 | ARID,HMG_box | 158 | 64 | 84 | 60 |
| 5 | ARCH5 | RBB1NT,ARID,  Tudorknot | 150 | 26 | 87 | 25 |
| 6 | ARCH7 | ARID,HSP20 | 97 | 26 | 88 | 23 |
| 7 | ARCH9 | ARID, RFX_DNA_binding | 68 | 30 | 83 | 26 |
| 8 | ARCH12 | BRCT_2, Myb_DNAbind_2, ARID,Rap1_C | 31 | Not clustered | 85 | 31 |
| 9 | ARCH20 | ARID,Med15_fungi | 13 | Not clustered | 92 | 13 |
| 10 | ARCH30 | Myb_DNAbind_2, ARID,Rap1_C | 6 | Not clustered | 87 | 6 |
| 11 | ARCH37 | DNMT1RFD,ARID | 4 | Not clustered | 83 | 4 |
