# Supplementary material for: Domain architecture of BAF250a reveals the ARID and ARM-repeat domains with implication in function and assembly of the BAF remodeling complex
Source: PLoS One. 2018 Oct 11;13(10):e0205267. doi: 10.1371/journal.pone.0205267 (PMC6181354; doi:10.1371/journal.pone.0205267)
Supplement: S2 Table — If particular residue is found to be conserved, single letter amino acid code is written in bracket next to the biochemical nature of the amino acids. * Marked indicate the position that is selectively conserved in ARID in ARCH2 as compared to all other architectures. DNA binding residues from previous study (reference # 19 in the main text) were highlighted in bold face. Therefore, functionality of the uniquely conserved residues (*) may be attributed as DNA binding where it is written in bold. (DOCX) [file pone.0205267.s009.docx]

**S2 Table. Biochemical property-based conservation at 70% level for each residues in all ARID across 11 different domain architectures vs. ARID in ARCH2 (BAF250a ARID, PDB ID: 1RYU).** If particular residue is found to be conserved, single letter amino acid code is written in bracket next to the biochemical nature of the amino acids. * Marked indicate the position that is selectively conserved in ARID in ARCH2 as compared to all other architectures. DNA binding residues from previous study (reference # 19 in the main text) were highlighted in bold face. Therefore, functionality of the uniquely conserved residues (*) may be attributed as DNA binding where it is written in bold.

| **Residue in 1RYU** | **Biochemical property-based conservation (70%) at corresponding residue position in ARID across all domain architectures** | **Biochemical property-based conservation (70%) at corresponding residue position in all ARID within architecture 2 (ARID and BAF250_C)** | **All atom relative accessibilities (%)** |
| --- | --- | --- | --- |
| *1019E | Variable | Conserved  (E) | 54.1 |
| 1020R | Polar | Polar (R) | 29.7 |
| *1021K | Variable | Conserved (basic) | 32.7 |
| 1022M | Variable | Variable | 59.4 |
| 1023W | Aromatic hydrophobic | Aromatic hydrophobic | 25.7 |
| 1024V | Hydrophobic | Hydrophobic (aliphatic) | 1 |
| 1025D | Polar | Polar (acidic) | 50.6 |
| *1026R | Variable | Conserved (basic) | 55.3 |
| 1027Y | Hydrophobic | Hydrophobic | 4.4 |
| *1028L | Variable | Conserved (aliphatic hydrophobic) | 3.3 |
| 1029A | Polar | Polar | 49.2 |
| 1030F | Hydrophobic | Hydrophobic (F) | 44.3 |
| 1031T | Hydrophobic | Hydrophobic | 2 |
| 1032E | Polar | Polar (E) | 47.5 |
| *1033E | Variable | Conserved (charged) | 76.5 |
| *1034K | Variable | Conserved (basic) | 48.8 |
| 1035A | Small | Small | 77.5 |
| *1036M | Variable | Conserved (polar) | 37.1 |
| 1037G | Small | Small (P) | 10.6 |
| 1038M | Hydrophobic | Hydrophobic (aliphatic) | 81.6 |
| 1049T | Polar | Polar | 72 |
| 1040N | Variable | Variable | 20.7 |
| ***1041L** | Variable | Conserved (hydrophobic) | 23.1 |
| 1042P | P | P | 0.5 |
| 1043A | Variable | Variable | 0.2 |
| **1044V** | Hydrophobic | Hydrophobic (aliphatic) | 2.3 |
| 1045G | Variable | Variable | 46.5 |
| ***1046R** | Variable | Conserved (K) | 59 |
| **1047K** | Polar | Polar | 42.2 |
| 1048P | Polar | Small | 14.5 |
| 1049L | Aliphatic hydrophobic | L | 0.1 |
| 1050D | Polar | D | 2.8 |
| 1051L | L | L | 2.2 |
| 1052Y | Aromatic hydrophobic | Aromatic hydrophobic | 28.8 |
| 1053R | Polar | Basic | 47.6 |
| 1054L | L | L | 1.1 |
| 1055Y | Aromatic hydrophobic | Y | 12.5 |
| *1056V | Variable | Aliphatic hydrophobic | 43.2 |
| 1057S | variable | variable | 12.6 |
| 1058V | V | V | 5.1 |
| *1059K | Variable | Conserved (basic) | 57.2 |
| 1060E | Polar | Acidic | 84 |
| 1061I | Hydrophobic | Hydrophobic | 14.8 |
| 1062G | G | G | 62.3 |
| 1063G | G | G | 19.5 |
| 1064L | Hydrophobic | Hydrophobic | 41 |
| 1065T | Polar | Variable | 78.6 |
| 1066Q | Conserved (polar) | Conserved (polar) | 43.5 |
| 1067V | V | V | 0.4 |
| 1068N | Small | Polar | 62.6 |
| 1069K | Polar | K | 72.4 |
| 1070N | Polar | Polar | 45.2 |
| 1071K | K | K | 60.6 |
| ***1072K** | Variable | Polar | 20.5 |
| **1073W** | W | W | 31.2 |
| ***1074R** | Variable | Conserved (basic) | 46 |
| 1075E | Polar | Acidic | 45.2 |
| 1076L | Hydrophobic | Aliphatic hydrophobic | 0.1 |
| 1077A | Hydrophobic | Hydrophobic | 0 |
| *1078T | Variable | Small | 56.8 |
| 1079N | Variable | Variable | 57.7 |
| 1080L | Hydrophobic | Hydrophobic | 5.5 |
| *1081N | Variable | Conserved (polar) | 55.7 |
| 1082V | Hydrophobic | Hydrophobic (aliphatic) | 18.1 |
| 1083G | Small | Small | 49 |
| *1084T | Variable | Small | 38 |
| *1085S | Variable | Conserved (S) | 67.4 |
| 1086S | Polar | Polar | 70.4 |
| *1087S | Variable | Conserved (S) | 73.9 |
| 1088A | A | A | 10 |
| 1089A | Small | Small (bend like) | 5.6 |
| 1090S | Hydrophobic | Variable | 65.1 |
| ***1091S** | Variable | Polar | 25.5 |
| **1092L** | Hydrophobic | L | 1.8 |
| **1093K** | Basic | Basic | 22.1 |
| ***1094K** | Variable | Conserved (K) | 59.3 |
| *1095Q | Variable | Conserved (polar) | 17.8 |
| **1096Y** | Y | Y | 2.9 |
| ***1097I** | Variable | Conserved (hydrophobic) | 35.7 |
| 1098Q | Polar | Polar | 45.3 |
| 1099C | Hydrophobic | Hydrophobic | 0.4 |
| 1100L | L | L | 1.2 |
| 1101Y | Hydrophobic | Hydrophobic | 69.7 |
| *1102A | Variable | Conserved (small) | 61.3 |
| 1103F | Aromatic hydrophobic | Aromatic hydrophobic | 2.7 |
| 1104E | E | E | 17.3 |
