# Supplementary material for: Domain architecture of BAF250a reveals the ARID and ARM-repeat domains with implication in function and assembly of the BAF remodeling complex
Source: PLoS One. 2018 Oct 11;13(10):e0205267. doi: 10.1371/journal.pone.0205267 (PMC6181354; doi:10.1371/journal.pone.0205267)
Supplement: S3 Table — Only mutations that map to the ARID and BAF250_C terminal domains are listed here. (DOCX) [file pone.0205267.s010.docx]

**S3 Table. List of missense mutations reported for the BAF250a in the COSMIC database.** Only mutations that map to the ARID and BAF250_C terminal domains are listed here.

| **Missense mutations in ARID domain** | | |
| --- | --- | --- |
| **Position** | **Mutation** | **Mutation (AA)** |
| 1004 | c.3010A>G | p.T1004A |
| 1013 | c.3037G>A | p.E1013K |
| 1017 | c.3049G>A | p.E1017K |
| 1020 | c.3059G>A | p.R1020K |
| 1020 | c.3060G>T | p.R1020S |
| 1022 | c.3064A>G | p.M1022V |
| 1023 | c.3068G>T | p.W1023L |
| 1026 | c.3076C>T | p.R1026C |
| 1027 | c.3080A>G | p.Y1027C |
| 1045 | c.3133G>A | p.G1045S |
| 1046 | c.3137G>A | p.R1046K |
| 1048 | c.3142C>T | p.P1048S |
| 1049 | c.3145C>G | p.L1049V |
| 1050 | c.3148G>A | p.D1050N |
| 1051 | c.3152T>G | p.L1051R |
| 1053 | c.3158G>A | p.R1053H |
| 1055 | c.3163T>C | p.Y1055H |
| 1055 | c.3164A>G | p.Y1055C |
| 1063 | c.3188G>T | p.G1063V |
| 1066 | c.3197A>G | p.Q1066R |
| 1074 | c.3220C>T | p.R1074W |
| 1074 | c.3221G>A | p.R1074Q |
| 1077 | c.3229G>A | p.A1077T |
| 1077 | c.3230C>G | p.A1077G |
| 1080 | c.3238C>A | p.L1080I |
| 1083 | c.3247G>T | p.G1083C |
| 1090 | c.3269G>T | p.S1090I |
| 1100 | c.3299T>G | p.L1100R |
| 1106 | c.3318G>T | p.K1106N |
| 1107 | c.3319A>G | p.I1107V |
| 1109 | c.3325C>T | p.R1109W |
| 1110 | c.3329G>A | p.G1110E |
| 1112 | c.3334G>C | p.D1112H |
| 1123 | c.3368C>T | p.S1123F |
|  |  |  |
| **Missense mutations in BAF250_C** | | |
| **Position** | **Mutation** | **Mutation (AA)** |
| 1940 | c.5818C>T | p.P1940S |
| 1940 | c.5819C>T | p.P1940L |
| 1942 | c.5825G>A | p.G1942D |
| 1947 | c.5841G>T | p.Q1947H |
| 1950 | c.5848C>T | p.R1950W |
| 1950 | c.5849G>A | p.R1950Q |
| 1950 | c.? | p.R1950Q |
| 1958 | c.5872G>A | p.E1958K |
| 1958 | c.? | p.E1958K |
| 1961 | c.5882G>A | p.S1961N |
| 1965 | c.5894C>T | p.T1965I |
| 1969 | c.5906C>T | p.T1969I |
| 1981 | c.5941T>C | p.C1981R |
| 1982 | c.5944G>A | p.V1982I |
| 1982 | c.? | p.V1982I |
| 1985 | c.5953T>C | p.S1985P |
| 1986 | c.5958T>G | p.N1986K |
| 1989 | c.5966G>T | p.R1989L |
| 1992 | c.5974T>C | p.S1992P |
| 1992 | c.5975C>T | p.S1992L |
| 1996 | c.5987G>A | p.G1996D |
| 1997 | c.5989A>G | p.N1997D |
| 1998 | c.5993A>G | p.D1998G |
| 2000 | c.5998G>C | p.E2000Q |
| 2000 | c.5999A>T | p.E2000V |
| 2002 | c.6005C>T | p.S2002F |
| 2008 | c.6023T>G | p.L2008R |
| 2015 | c.6043A>T | p.I2015F |
| 2018 | c.6052C>T | p.H2018Y |
| 2024 | c.6071G>A | p.R2024Q |
| 2025 | c.6074A>C | p.K2025T |
| 2032 | c.6094G>A | p.E2032K |
| 2035 | c.6103G>A | p.E2035K |
| 2042 | c.6125G>A | p.S2042N |
| 2046 | c.? | p.V2046L |
| 2050 | c.6148T>G | p.W2050G |
| 2064 | c.6191T>A | p.L2064H |
| 2064 | c.6191T>C | p.L2064P |
| 2066 | c.6196A>G | p.N2066D |
| 2068 | c.6203C>G | p.S2068W |
| 2068 | c.6203C>T | p.S2068L |
| 2070 | c.6210G>T | p.Q2070H |
| 2075 | c.6223_6224CC>TT | p.P2075L |
| 2079 | c.6236G>T | p.S2079I |
| 2083 | c.6248C>A | p.P2083H |
| 2084 | c.6251T>A | p.V2084D |
| 2084 | c.6251T>G | p.V2084G |
| 2087 | c.6259G>A | p.G2087R |
| 2087 | c.6260G>A | p.G2087E |
| 2087 | c.? | p.G2087R |
| 2088 | c.6263T>A | p.L2088H |
| 2089 | c.6265C>A | p.L2089I |
| 2089 | c.6266T>C | p.L2089P |
| 2090 | c.6270C>G | p.H2090Q |
| 2093 | c.6277G>T | p.V2093F |
| 2097 | c.6289G>T | p.A2097S |
| 2100 | c.6299A>G | p.Q2100R |
| 2101 | c.6301G>A | p.D2101N |
| 2102 | c.6305C>A | p.P2102H |
| 2103 | c.6307T>C | p.F2103L |
| 2104 | c.6310T>C | p.S2104P |
| 2104 | c.6311C>T | p.S2104F |
| 2105 | c.6313A>C | p.T2105P |
| 2105 | c.6314C>T | p.T2105I |
| 2106 | c.6316C>A | p.L2106M |
| 2113 | c.6338C>T | p.S2113F |
| 2114 | c.6341C>T | p.P2114L |
| 2123 | c.6368G>C | p.S2123T |
| 2127 | c.6381C>G | p.I2127M |
| 2131 | c.6393T>G | p.N2131K |
| 2135 | c.6403A>G | p.I2135V |
| 2135 | c.6404T>A | p.I2135N |
| 2138 | c.6413C>A | p.T2138K |
| 2139 | c.6416C>A | p.P2139H |
| 2143 | c.6427C>T | p.R2143C |
| 2143 | c.6428G>A | p.R2143H |
| 2157 | c.6469G>A | p.D2157N |
| 2158 | c.6473G>A | p.R2158Q |
| 2161 | c.6481C>A | p.P2161T |
| 2163 | c.6487T>C | p.C2163R |
| 2164 | c.6491G>T | p.R2164L |
| 2165 | c.6493G>C | p.E2165Q |
| 2171 | c.6512T>C | p.L2171P |
| 2177 | c.6529G>A | p.G2177R |
| 2179 | c.6536G>T | p.S2179I |
| 2188 | c.6563A>G | p.Q2188R |
| 2188 | c.6564G>T | p.Q2188H |
| 2191 | c.6572G>T | p.S2191I |
| 2195 | c.6584T>C | p.L2195P |
| 2197 | c.6590G>T | p.G2197V |
| 2198 | c.6593T>C | p.F2198S |
| 2200 | c.6598G>A | p.E2200K |
| 2202 | c.6604A>G | p.S2202G |
| 2202 | c.6605G>T | p.S2202I |
| 2204 | c.6611C>T | p.A2204V |
| 2206 | c.6617C>T | p.T2206I |
| 2210 | c.6630G>C | p.Q2210H |
| 2211 | c.6632G>A | p.S2211N |
| 2214 | c.6641G>C | p.S2214T |
| 2216 | c.6646C>T | p.L2216F |
| 2220 | c.6659A>G | p.N2220S |
| 2229 | c.6685G>T | p.D2229Y |
| 2232 | c.6694C>T | p.R2232W |
| 2233 | c.? | p.R2233W |
| 2236 | c.6706C>T | p.R2236C |
| 2236 | c.6707G>C | p.R2236P |
| 2236 | c.? | p.R2236C |
| 2237 | c.6709G>T | p.A2237S |
| 2239 | c.6716T>C | p.L2239P |
| 2240 | c.6718G>A | p.A2240T |
| 2244 | c.6731T>G | p.V2244G |
| 2255 | c.6763G>A | p.E2255K |
| 2255 | c.6765A>C | p.E2255D |
| 2257 | c.6770G>A | p.R2257Q |
| 2258 | c.6773T>G | p.L2258R |
| 2262 | c.6785C>T | p.S2262L |
| 2263 | c.6788T>C | p.V2263A |
| 2269 | c.6806C>T | p.S2269L |
| 2275 | c.6824T>C | p.I2275T |
