# Supplementary material for: Domain architecture of BAF250a reveals the ARID and ARM-repeat domains with implication in function and assembly of the BAF remodeling complex
Source: PLoS One. 2018 Oct 11;13(10):e0205267. doi: 10.1371/journal.pone.0205267 (PMC6181354; doi:10.1371/journal.pone.0205267)
Supplement: S1 Text — (DOCX) [file pone.0205267.s011.docx]

**Supporting Materials and methods**

**Cloning and expression of BAF250_C domain of BAF250A**

BAF250_C (residues 1926-2181) of BAF250a was cloned between NdeI and EcoRI restriction sites in pET28a vector. The protein contains an N-terminal 6XHis-tag. E. coli BL21(DE3)-Rosetta competent cells were transformed with the plasmid and cells were cultured in LB medium containing 50 mg/ml Kanamycin and 30 mg/ml chloramphenicol. Protein expression was optimized for the isopropyl-d-1-thiogalactopyranoside (IPTG) concentration and temperature. Best expression was achieved by using 0.5 mM IPTG for induction at 26°C for 8 h. Cells were harvested by centrifugation at 5000 rpm for 15 min and the pellet was resuspended in re-suspension buffer (50 mM Tris, pH 8, 500 mM NaCl, 10% Glycerol). The first step of purification involved Ni-NTA affinity chromatography. After sonication, the cell lysate was centrifuged (14000 rpm) for 1 h at 4°C and the supernatant was loaded onto Ni^2+^–NTA column. The column was washed with re-suspension buffer followed by wash buffer (50 mM Tris, pH 8, 500 mM NaCl, 20 mM imidazole, 10% Glycerol). The protein was eluted using elution buffer (50 mM Tris, pH 8, 500 mM NaCl, 250 mM imidazole, 10% Glycerol). Different fractions were collected and ran on SDS polyacrylamide gel to check the purity level of sample.

**Western blotting**

Different Fractions of Ni^2+^–NTA column purification were used for Western blotting. Bradford reagent (BIO-RAD Cat# 500-006) was used to perform protein quantification. Protein samples were normalized for total protein content and resolved on SDS polyacrylamide gel (SDS-PAGE) followed by overnight transfer to a PVDF (Polyvinylidene fluoride) membrane (GE, Cat#10600023) at 25V. The membrane was then blocked with a solution of 5% non-fat dried milk in TBST buffer (25 mM Tris-HCl, pH 7.5, 150 mM NaCl, 0.05% Tween 20) for 1 h at room temperature, further incubated with anti-His tag primary antibody (1:1000 dilutions for anti-His tag antibody from CUSABIO CAT# CSB-MA000159) overnight at 4°C. Membrane was washed 3 times with TBST to remove non-specifically bound primary antibody followed by peroxidase-conjugated secondary antibody (1:10000 dilution was used for secondary antibody from Santa Cruz, #sc2005) incubation for 1 hour at room temperature. Non-specifically bound secondary antibody was removed by washing the blot 3 times with TBST buffer. Signals were detected using chemiluminescence substrate solution (Thermo fisher # 34080) in gel documentation system (BioRad chemiDoc touch) and analyzed by a GE LAS4000 luminescent image analyzer (Fuji Photo Film).

**Supporting Results**

BAF250_C was best expressed using 0.5 mM IPTG for induction at 26°C for 8 h in *E. coli* BL21(DE3) cells (S7A Fig). We made attempts to purify protein from 2L LB culture and Ni^2+^–NTA affinity chromatography. Different fractions were collected after the Ni^2+^–NTA affinity column and run on SDS polyacrylamide gel. Most of the protein was expressed in the insoluble inclusion bodies and therefore only a very small fraction of the protein was eluted from the Ni^2+^–NTA column (S7B and S7C Figs). The presence and identity of the protein was confirmed by SDS-PAGE and Western blot analysis. Western Blot showed that the protein undergoes degradation in in the eluted fraction. Further optimization of the protein construct, expression conditions, and purification strategies will be required to purify protein in amount sufficient for further biochemical and structural studies.
